# Evolution of habitat preference in 243 species of Bent‐toed geckos (Genus *Cyrtodactylus* Gray, 1827) with a discussion of karst habitat conservation

**DOI:** 10.1002/ece3.6961

**Published:** 2020-11-22

**Authors:** L. Lee Grismer, Perry L. Wood, Minh Duc Le, Evan S. H. Quah, Jesse L. Grismer

**Affiliations:** ^1^ Herpetology Laboratory Department of Biology La Sierra University Riverside CA USA; ^2^ Department of Biological Sciences & Museum of Natural History Auburn University Auburn AL USA; ^3^ Department of Environmental Ecology Faculty of Environmental Sciences University of Science Vietnam National University, Hanoi Hanoi Vietnam; ^4^ Central Institute of Natural Resources and Environmental Studies Vietnam National University, Hanoi Hanoi Vietnam; ^5^ Department of Herpetology American Museum of Natural History New York NY USA; ^6^ Institute of Tropical Biodiversity and Sustainable Development Universiti Malaysia Terengganu Terengganu Malaysia

**Keywords:** ancestral state reconstruction, Asia, ecology, Gekkonidae, limestone, phylogeny, stochastic character mapping

## Abstract

Understanding the processes that underpin adaptive evolutionary shifts within major taxonomic groups has long been a research directive among many evolutionary biologists. Such phenomena are best studied in large monophyletic groups that occupy a broad range of habitats where repeated exposure to novel ecological opportunities has happened independently over time in different lineages. The gekkonid genus *Cyrtodactylus* is just such a lineage with approximately 300 species that range from South Asia to Melanesia and occupy a vast array of habitats. Ancestral state reconstructions using a stochastic character mapping analysis of nine different habitat preferences were employed across a phylogeny composed of 76% of the known species of *Cyrtodactylus*. This was done in order to ascertain which habitat preference is the ancestral condition and from that condition, the transition frequency to more derived habitat preferences. The results indicate that a general habitat preference is the ancestral condition for *Cyrtodactylus* and the frequency of transitioning from a general habitat preference to anything more specialized occurs approximately four times more often than the reverse. Species showing extreme morphological and/or ecological specializations generally do not give rise to species bearing other habitat preferences. The evolution of different habitat preferences is generally restricted to clades that tend to occur in specific geographic regions. The largest radiations in the genus occur in rocky habitats (granite and karst), indicating that the transition from a general habitat preference to a granite or karst‐dwelling life style may be ecologically uncomplicated. Two large, unrelated clades of karst‐associated species are centered in northern Indochina and the largest clade of granite‐associated species occurs on the Thai‐Malay Peninsula. Smaller, independent radiations of clades bearing other habitat preferences occur throughout the tree and across the broad distribution of the genus. With the exception of a general habitat preference, the data show that karst‐associated species far out‐number all others (29.6% vs. 0.4%–10.2%, respectively) and the common reference to karstic regions as “imperiled arcs of biodiversity” is not only misleading but potentially dangerous. Karstic regions are not simply refugia harboring the remnants of local biodiversity but are foci of speciation that *continue* to generate the most speciose, independent, radiations across the genus. Unfortunately, karstic landscapes are some of the most imperiled and least protected habitats on the planet and these data continue to underscore the urgent need for their conservation.

## Graphical abstract

The gekkonid genus is an ecologically labile lineage. Species with a general habitat preference have independently given rise to at least eight other distinct more specialized habitat preferences.

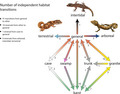

## 1 INTRODUCTION

The proliferation of phylogenetic and ecological diversity often results from exposure to novel ecological situations that provide ancestral species opportunities to shift particular aspects of their life style in order to adapt to different environments (Schluter, 2000; Losos, 2009; Glor, 2010; Pincheira‐Donoso et al., 2015; but see Wainwright & Price, 2016). Multiple lineages of species within a larger lineage may converge ecologically and morphologically when the closely related ancestral species of those lineages are exposed to the same novel set of ecological opportunities. As such, they may independently acquire the same morphological and ecological key innovations allowing them to exploit previously unoccupied niches (Losos, 2010; Pincheira‐Donoso et al., 2015; Pincheira‐Donoso & Meiri, 2013; Yoder et al., 2010). Understanding the processes that underpin these adaptive shifts are best studied in large monophyletic groups adapted to a broad range of habitats where repeated exposure to novel ecological opportunities has happened independently over time in different lineages on the same phylogenetic tree (e.g., Chiba, 2004; Genner et al., 2007; Gillespie, 2004; Harvey & Pagel, 1991; Landry et al., 2007; Losos, 2009; Skinner et al., 2008; Williams, 1983). This enables researchers to decipher between ecological and morphological similarities based on common ancestry versus those generated independently by similar selection pressures in similar environments on a similar body plan and genetic constitution (e.g., Baxter et al., 2008; Chan et al., 2010; Gross et al., 2009; Mahler et al., 2013; Toyama, 2017).

**FIGURE 1:**
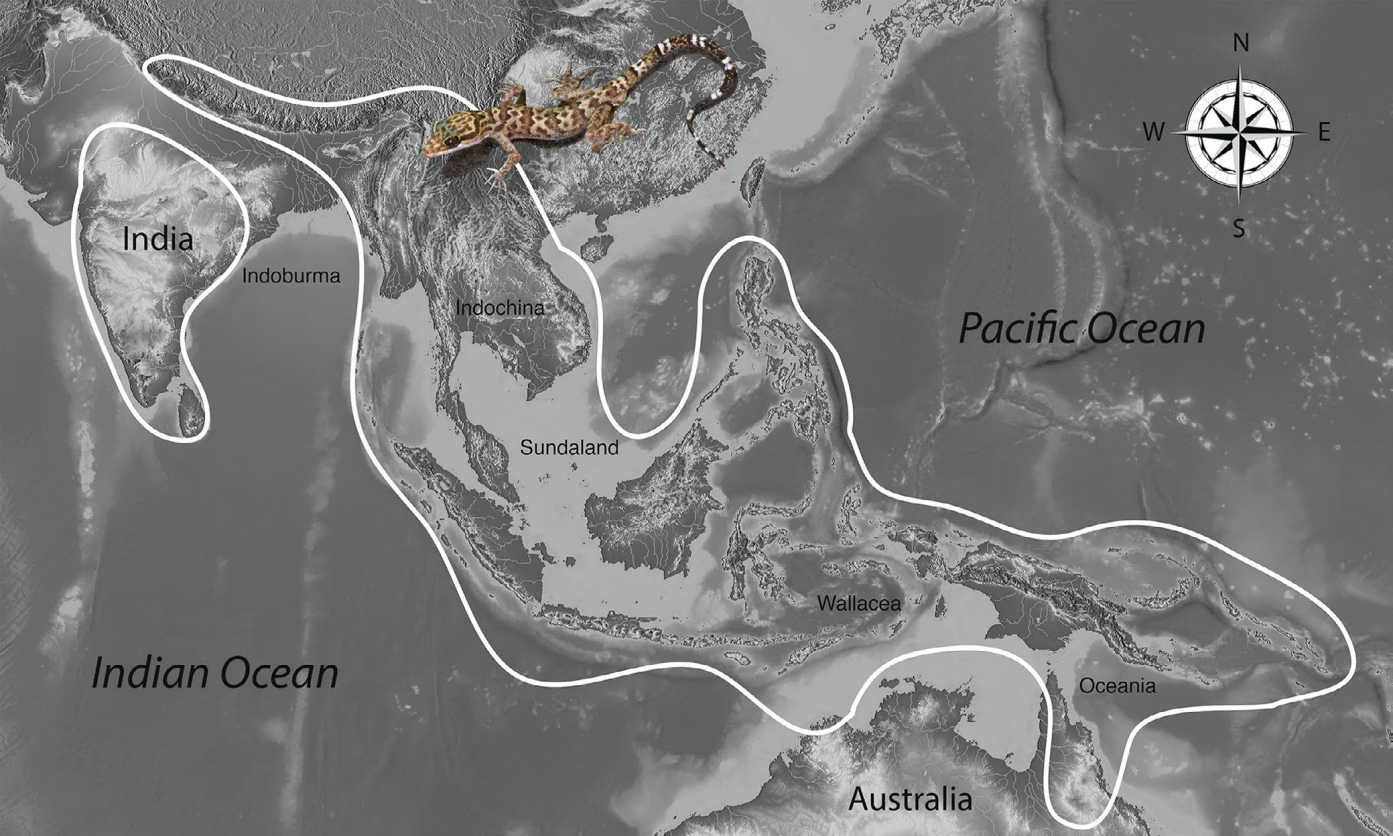
Distribution of the genus *Cyrtodactylus*

**FIGURE 2:**
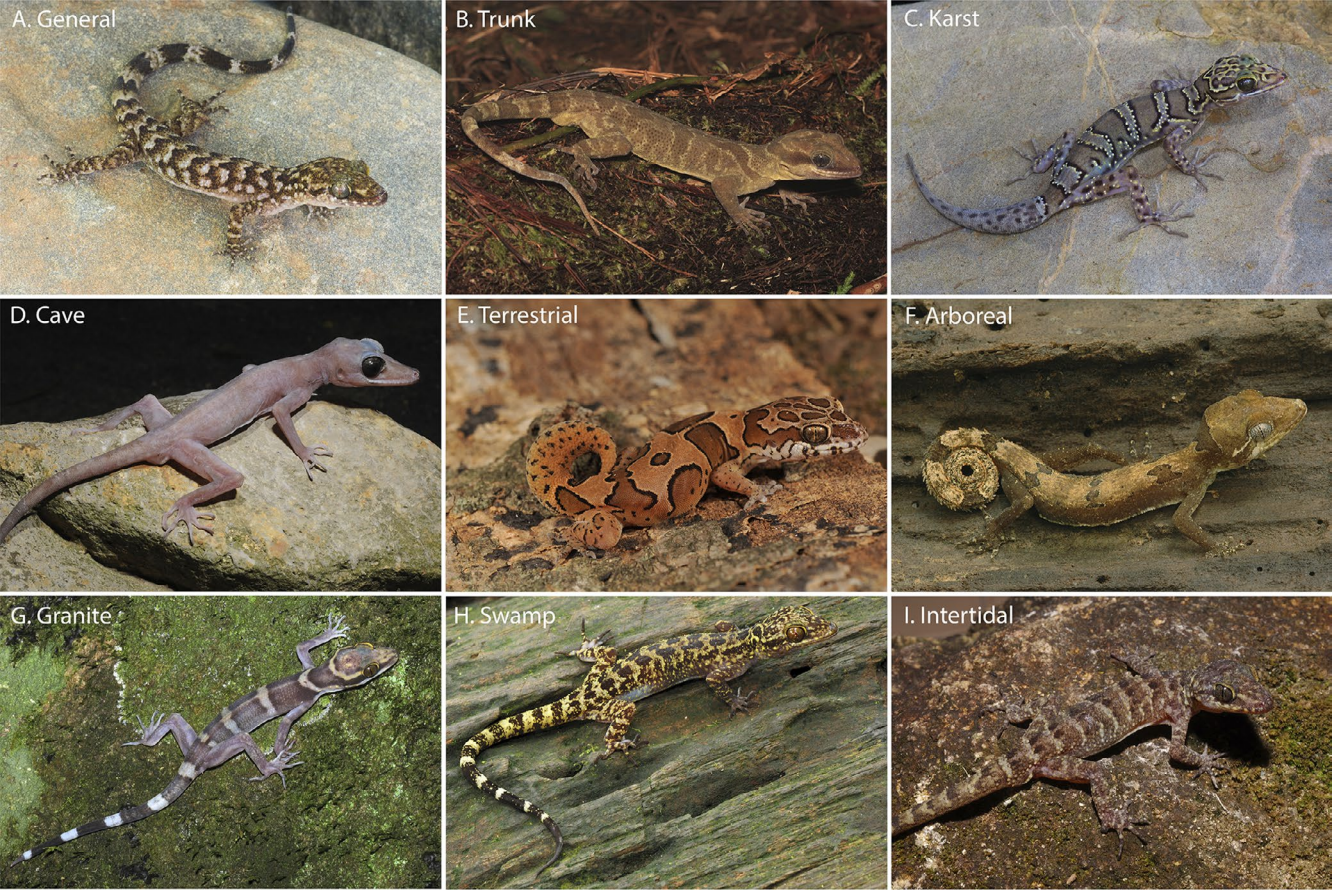
Representative species of the ecotypes associated with the habitat preferences delineated in this study. (a) *Cyrtodactylus mombergi*, (b) *C. solomonensis*, (c) *C. linnwayensis*, (d) *C. nigriocularis*, (e) *C. srilekhae*, (f) *C. elok*, (g) *C. tiomanensis*, (h) *C. pantiensis*, and (i) *C. seribuatensis*. Photos: a., c., f., g., h., i., L. Lee Grismer; b. Scott Travers; d. Nickolay A. Poyarkov; and e. Ishan Agarwal

In the last decade, the remarkable increase in the discovery of new species in the ecologically and morphologically diverse gekkonid genus *Cyrtodactylus* Gray, 1827 (see Grismer et al., 2020a) makes it a promising model to study the evolution of adaptive shifts in their habitat preference. *Cyrtodactylus* currently contains 296 nominal species (as of 10 July 2020; Uetz et al., 2020) that collectively range from South Asia to Melanesia (Figure 1). As would be expected from a group this large and widely distributed, it bears a broad variety of ecotypes (Figure 2) ranging from terrestrial to arboreal to cave‐dwelling and substrate specialists (e.g., Grismer & Grismer, 2017; Grismer, Wood, Thura, Zin, et al., 2018; Hikida, 1990; Johnson et al., 2012; Kraus, 2008; Welton et al., 2010; Youmans & Grismer, 2006; Figure 2). Yet despite this ecological diversity, the vast amount of research on this group pertains to descriptive taxonomic, phylogenetic, and biogeographic studies and there are relatively few detailed studies pertaining to life history and ecology (e.g., Aksornneam et al., 2019; Loos et al., 2010; Riedel et al., 2020). Given that *Cyrtodactylus* ranges across some of the most biologically diverse (and imperiled) landscapes in Asia, it plays multiple roles throughout a wide range of ecosystems similar to that of ecologically diverse, radiations of New World iguanian lizards (Losos, 2009; Mahler et al., 2013; Pincheira‐Donoso et al., 2015; Toyama, 2017; Williams, 1983). As such, understanding *Cyrtodactylus* ecology—even to a limited extent—and the evolution of habitat preference could provide insights into the evolution of their varied natural histories and could be foundational to future hypotheses of related comparative studies. Additionally, such data could directly impact the conservation and management of the ecosystems they inhabit.

Wilmer and Couper (2015) were the first to examine the evolution of habitat preference using 68 species of *Cyrtodactylus* based on the genus‐wide phylogeny of Wood et al. (2012) using Maximum Parsimony (MP) and Maximum Likelihood (ML) frameworks. Their analyses primarily focused on habitat shifts among species in the Papua Clade (sec. Wood et al., 2012) using five, broadly constructed habitat categories. In this study, we generated a phylogeny with 243 species (218 named and 25 undescribed) representing the most complete phylogeny for *Cyrtodactylus* with 76% of all species represented. Therefore, with an increase of 228 species to the data set now representing all major clades delimited in Wood et al. (2012), Grismer, Wood, Thura, Zin, et al. (2018), and O'Connell et al. (2019); nine finely delimited habitat preference categories as opposed to five; and a more informative statistical model (Bayesian vs. MP and ML; see below), we evaluate the evolution of habitat preference across the whole of *Cyrtodactylus*. With these data, we aim to (a) investigate the degree of ecological lability within *Cyrtodactylus* by assessing the frequency and direction of habitat preference transformations, (b) note the degree of convergent evolution in habitat preference among species and clades, (c) identify the habitats that support the largest number of species and, (d) contextualize these results in the framework of karst habitat conservation. These issues will be addressed using a stochastic character mapping analysis that that will map the most probable ancestral habitat preference at each node on the tree.

## 2 METHODS

### 2.1 Habitat preferences and ecotypes

*Cyrtodactylus* is by far the most speciose and ecologically diverse gekkotan genus (Uetz et al., 2020). We identified nine major habitat preferences and their associated ecotypes we believe can be justifiably utilized for the species in this analysis (Figure 2; Table S1). Habitat preference for each species was coded as a discrete character state derived from varying combinations of data from the literature, firsthand experience of the authors, and personal communication with researchers familiar with particular species. Although some of these categories could be further subdivided (e.g., arboreal into branch, twig, leaf, etc.), the subdivisions become less defensible owing to a lack of detailed microhabitat information for most species. Unfortunately, in this regard, most species can be considered data‐deficient, being that baseline information on their ecological requirements is limited to anecdotal observations or statements made in species descriptions based on limited contact at the time of their collection (e.g., Grismer, Wood, Thura, Zin, et al., 2018). We are aware of the potential biases of using limited observations from a single locality as representing the habitat preference of an entire species. However, in many cases, these are the only data available. Nonetheless, judiciously vetted, anecdotal natural history observations summarized across the literature coupled with our own field observations and those of others can provide a useful framework for supporting robust, testable, downstream hypotheses regarding the evolution of habitat preference. The habitat preferences and their associated ecotypes bearing the same categorical names are described below. Obvious morphological correlates associated with some ecotypes are noted only for additional clarity.

*General* (Figure 2a). Species that utilize all or nearly all of the microhabitats in their immediate surroundings in whatever environment they may inhabit (i.e., arid or tropical). The microhabitats may include rocks of all types (when present), logs, tree trunks, and all vegetative structures of various dimensions, as well as the ground in many cases. No particular microhabitat is notably preferred over any other although some species may be most common in low vegetation.

*Arboreal* (Figure 2f). These are relatively small, cryptically colored species (Grismer, 2011; Harvey et al., 2016) generally restricted to small branches, leaves, trunks of varying sizes, and shrubs. Some species may take refuge beneath exfoliating bark often as high or higher than three meters above the ground (Ellis & Pauwels, 2012). These species are rarely observed on the ground or lower than 1.5 m above the ground. In such instances, it is usually during windy and/or rainy nights (perhaps forced down from higher up; Dring, 1979; authors personal observation). All species have a prehensile tail used as a climbing aid (Dring, 1979; Grismer, 2008; Harvey et al., 2016) and usually carried in a coiled, elevated position.

*Trunk* (Figure 2b). These are species generally found on the trunks and large branches of large trees at varying heights that often take refuge in cracks, crevices, or holes in the trunks. They may occasionally occur on large granite rocks but only if the rocks are near the trees. These species are generally the largest and most robust species in the genus (Nielsen & Oliver, 2017; Oliver et al., 2014, 2016).

*Karst* (Figure 2c). These are generally more gracile species that are restricted to habitats where limestone rock (karst) is present. Individuals utilize this substrate (including cliff faces, small rocks, and boulders) as well as adjacent vegetation. If caves are present, they will enter only into the twilight zone and usually no deeper than 50 m (Grismer, Wood, Thura, Zin, et al., 2018). Despite what has been written about many karst‐associated species being cave species (e.g. Ellis & Pauwels, 2012), none are truly cave‐adapted and all are found on the outside of caves as well (see below). These species do not occur in habitats lacking karstic substrates.

*Granite* (Figure 2g). These are generally more robust species that are found in forested habitats bearing large granite boulders (not just small, scattered, granite rocks). Vegetation may be utilized but individuals occur more commonly on the granite boulders in all plains of orientation. These species do not occur in forested areas lacking granite boulders.

*Cave* (Figure 2d). These are species that occur exclusively in the cave‐like environments resulting from large granite boulders piled on top of one another. These species rarely occur on the outside surfaces of the boulders (i.e., the forest‐side) and for the most part, are restricted to the spaces between the boulders at varying depths below the surface of the ground in extremely low levels of illumination. These are truly cave‐adapted species with notably thin, gracile bodies, long limbs, flat heads, large eyes, and faded color patterns (Grismer & Grismer, 2017; Ngo, 2008; Nguyen et al., 2006).

*Terrestrial* (Figure 2e). These are species that generally occur only on the ground. They may occasionally be found on the tops of small rocks (when present) or on the base of small trees and shrubs but never higher than 1 m above the ground. These species are relatively small and notably squat with short tails, heads, and digits (Agarwal, 2016; Grismer et al., 2019).

*Swamp* (Figure 2h). These are species that are restricted to swampy habitats and utilize low, viny vegetation, the trunks of small trees and shrubs, or small logs often above or in close proximity to water. These species do not have prehensile tails nor do they occur higher than 1 m above the ground as do arboreal species. Additionally, they are never found on the trunks of large trees nor do they take refuge in tree crevices as do trunk species. These species generally have large eyes with notably reddish‐orange irises (Grismer et al., 2012; Riyanto et al., 2015).

*Intertidal* (Figure 2i). This category contains a single species that occurs exclusively in the rocky intertidal zones of small islands in the Seribuat Archipelago off the southeast coast of Peninsular Malaysia and avoids nearby forested regions even if they lack other species of *Cyrtodactylus* (Youmans & Grismer, 2006). Even though they do not forage in vegetation, they have a generalized body structure vastly different from that of squat, stocky, terrestrial species.

### 2.2 Molecular data

A data set composed of 1,341 individuals of *Cyrtodactylus* was constructed from newly sequenced samples (*n* = 36) and GenBank (*n* = 1,305). In order to maximize species coverage and not gene coverage, we used a 1,454 base pair segment of only the mitochondrial gene NADH dehydrogenase subunit 2 (ND2) and its flanking tRNAs. The data set was pruned to include 243 individuals (Table S1) representing one individual per species (218 named and 25 unnamed—i.e., phylogenetically identified species in other publications that remain undescribed). *Hemidactylus frenatus* and *Mediodactylus russowii* were used as outgroups to root the tree following Gamble et al. (2012). Genomic DNA was isolated from liver or skeletal muscle from specimens stored in 95% ethanol using a SPRI magnetic bead extraction protocol (https://github.com/phyletica/lab‐protocols/blob/master/extraction‐spri.md). The ND2 gene was amplified using a double‐stranded Polymerase Chain Reaction (PCR) under the following conditions: 1.0 µl genomic DNA (10–30 µg), 1.0 μl (10 μM concentration) light strand primer (L4437b, 5ʹ–AAGCAGTTGGGCCCATRCC–3ʹ, Macey et al., 1997) 1.0 μl (10 μM concentration) heavy primer (H5934, 5ʹ–AGRGTGCCAATGTCTTTGTGRTT–3ʹ, Macey et al., 1997), 1.0 µl dinucleotide pairs (1.5 µM), 2.0 µl 5× buffer (1.5 µM), MgCl 10× buffer (1.5 µM), 0.1 µl Taq polymerase (5 u/µl), and 6.4 µl ultra‐pure H_2_O. PCR reactions were executed on Axygen Maxygene II gradient thermocycler under the following conditions: initial denaturation at 95°C for 2 min, followed by a second denaturation at 95°C for 35 s, annealing at 55°C for 35 s, followed by a cycle extension at 72°C for 35 s, for 31 cycles. All PCR products were visualized on a 1.0% agarose gel electrophoresis. Successful PCR products were sent to GENEWIZ^®^ for PCR purification, cycle sequencing, sequencing purification, and sequencing using the same primers as in the amplification step. Sequences were visualized from both the 3ʹ and the 5ʹ ends separately to confirm congruence between reads. Forward and reverse sequences were uploaded and edited in Geneious™ 2019.0.4 (https://www.geneious.com). Following sequence editing, alignment was accomplished using the MAFTT v7.017 (Katoh & Kuma, 2002) plugin under the default settings in Geneious™ 2019.0.4 (https://www.geneious.com ) and checked by eye. Mesquite v3.04 (Maddison & Maddison, 2015) was used to calculate the correct amino acid reading frame and to confirm the lack of premature stop codons.

### 2.3 Phylogenetic analyses

An ML analysis was implemented on the IQ‐TREE webserver (Nguyen et al., 2015; Trifinopoulos et al., 2016) preceded by the selection of substitution models using the Bayesian Information Criterion (BIC) in ModelFinder (Kalyaanamoorthy et al., 2017) that supported the GTR + F+I + G4 as the best fit model of evolution for codon positions 1 and 3, TIM + F+I + G4 for codon position 2, and GTR + F+I + G4 as the best fit model for the tRNAs. One‐thousand bootstrap pseudoreplicates via the ultrafast bootstrap approximation algorithm (UFB; Hoang et al., 2018) were employed, and nodes having ML UFB values of 95 and above were considered strongly supported (Minh et al., 2013). We considered UFB values of 90 and above to be well‐supported.

An input file implemented in BEAUti (Bayesian Evolutionary Analysis Utility) version 2.4.6 was run in BEAST (Bayesian Evolutionary Analysis Sampling Trees) version 2.4.6 (Drummond et al., 2012) on CIPRES (Cyberinfrastructure for Phylogenetic Research; Miller et al., 2010) in order to generate a BEAST phylogeny, employing a lognormal relaxed clock with unlinked site models and linked trees and clock models. bModelTest (Bouckaert & Drummond, 2017), implemented in BEAST, was used to numerically integrate over the uncertainty of substitution models while simultaneously estimating phylogeny using Markov chain Monte Carlo (MCMC). MCMC chains were run using a Yule prior for 125,000,000 million generations and logged every 12,500 generations. The BEAST log file was visualized in Tracer v. 1.6.0 (Rambaut et al., 2014) to ensure effective sample sizes (ESS) were above 200 for all parameters. A maximum clade credibility tree using mean heights at the nodes was generated using TreeAnnotator v.1.8.0 (Rambaut & Drummond, 2013) with a burnin of 1,000 trees (10%). Nodes with Bayesian posterior probabilities (BPP) of 0.95 and above were considered strongly supported (Huelsenbeck et al., 2001; Wilcox et al., 2002). We considered BPP values of 0.90 and above to be well‐supported.

To ascertain the potential effect of third codon saturation on tree topology, we constructed IQ‐TREE and BEAST trees using the same 243 species data set with the third codon deleted and following the same partition schemes outlined above. Additionally, ND2 topology and nodal support were compared to IQ‐TREE and BEAST trees generated from a nuclear data set derived from Wood et al. (2012) using their partition and outgroup scheme and the nuclear genes MXRA5 (839 bp), PDC (395 bp), and RAG1 (1,050 bp). The mitochondrial and nuclear genes were not concatenated since that would have resulted in a data set with approximately 55% of the data missing. Instead, we opted to maximize complete species coverage as opposed to incomplete gene coverage.

### 2.4 Ancestral state reconstruction

To avoid the arguable shortcomings associated with MP and ML ancestral state reconstructions (see Huelsenbeck et al., 2001), we used a more statistically robust Bayesian framework of stochastic character mapping (SCM; Revell, 2012) in order to derive probability estimates of the ancestral states of the habitat preferences at each node in the tree. We employed a SCM analysis implemented in R [v3.4.3] (R Core Team, 2018) using the R package Phytools (Revell, 2012) on the BEAST tree. To accommodate uncertainty in phylogenetic history, SCM averages all parameter values from a continuous‐time Markov chain of character state change that weights them in proportion to their posterior probability (i.e., their probability of occurrence at each node). We identified the transition rate matrix that best fit the data by comparing likelihood scores among alternate models using the Akaike Information Criterion (AIC) in the R package ape 5.2 (Paradis & Schilep, 2018). Three transition rate models were considered: a 72‐parameter model having different rates for every transition type (the ARD model); a 36‐parameter model with equal forward and reverse rates between states (the symmetrical rates SYM model); and a single rate parameter model that assumes equal rates among all transitions (ER). Lastly, an MCMC approach was used to sample the most probable 1,000 character histories from the posterior using *make.simmap*() and then summarized them using the *summary*() command.

## 3 RESULTS

**FIGURE 3:**
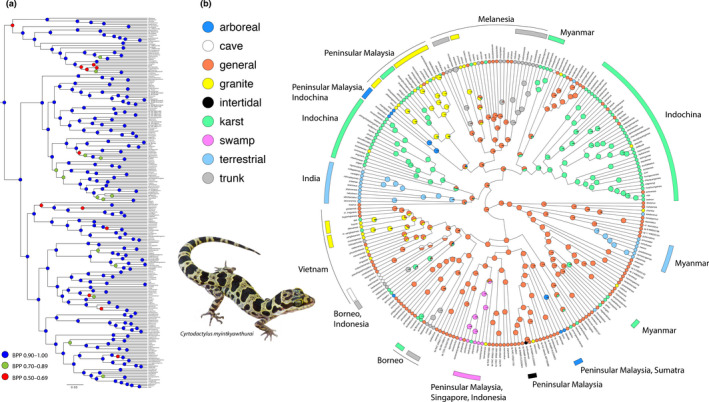
Maximum clade credibility BEAST phylogeny of the genus *Cyrtodactylus* with the range of nodal support values. B. Stochastic character map of nine habitat preferences on a maximum clade credibility BEAST phylogeny showing the probability of the ancestral habitat preference at each node, the independent origins of the same habitat preferences among clades and species and their geographic location, and the habitat preference of each species

The BEAST and IQ‐TREE analyses using all three ND2 codon positions recovered well‐supported trees with very similar topologies (Figure 3a; Figure S1). The IQ‐TREE analysis using just the first two codons recovered a tree whose topology does not closely match that of the three‐codon IQ‐TREE. Additionally, the two‐codon tree recovered poorly supported relationships not recovered in the three‐codon tree or trees derived from a number of more exclusive analyses of smaller clades. Furthermore, it is replete with short internodes, low branch support values, and polytomies (Figure S2). The two‐codon BEAST tree was even less informative with much of it being composed of large polytomies (Figure S2).

The nuclear IQ‐TREE amounted to little more than large polytomy lacking deep nodal support (Figure S1) and recovered a paraphyletic *Cyrtodactylus* by placing *C. tibetanus* within the outgroups. The nuclear BEAST tree performed slightly better but still had weak support at the deeper nodes, short internode branch lengths, and relationships not seen in other trees. As previously noted by Wilmer and Couper (2015), the use of nuclear markers did not change the support or increase the resolution at any of the deep nodes of the three‐codon trees. Based on these results, the complete data set of the three‐codon ND2 BEAST tree was the most complete, was very similar to the three‐codon ND2 IQ‐TREE, and performed best. As such, it was used for the SCM analysis.

The BEAST analysis recovered a tree with generally strong nodal support throughout (Figure 3a) and whose species relationships are largely consistent with those recovered in previous phylogenies more focused on smaller clades. The likelihood scores for the three transition rate models were ARD = −312.403; SYM = −275.474; and ER = −253.168. The SCM analysis using an ER model demonstrated that specific habitat preferences occur in clades from both general (large) and localized (small) geographic regions (Figure 3b). Most notably, it recovered a general habitat preference as being ancestral to all other preferences in having the highest probability of occurrence across all the deep nodes of the tree and most of the shallow nodes. The analysis recovered two large, independently evolved clades of karst species in Indochina, two independently evolved smaller clades in Myanmar, and another smaller clade on the Thai‐Malay Peninsula. Other individual karst species are scattered widely across the tree and across the distribution of the genus (Figure 3b). Independently evolved clades of terrestrial species occur in South Asia (India and Sri Lanka) and the Ayeyarwady Basin of Myanmar. A single swamp clade ranges across Peninsular Malaysia, Singapore, and Indonesia. Within Melanesia, two independently evolved clades of trunk species occur in Papua New Guinea. There are also independently evolved granite, karst, and trunk species that occur in other Melaneisan regions as well. Two smaller trunk clades evolved independently in Borneo and Indonesia. Two closely related clades of granite species occur in Peninsular Malaysia (out of which the Thai‐Malay Peninsula karst clade evolved) and another in Vietnam out of which two karst species independently evolved. Two small, distantly related clades of arboreal species evolved independently—one in Peninsular Malaysia and Indochina and the other in Peninsular Malaysia and Sumatra. There is a single cave species, *Cyrtodactylus nigriocularis* from southern Vietnam that has converged on a small clade of cave species also from southern Vietnam. A swamp species from Peninsular Malaysia, *C. seminanjungensis,* converges on a clade of swamp species from Peninsular Malaysia, Singapore, and Indonesia. Lastly, there is a single intertidal species, *C. seribuatensis* from an archipleago off the southeast coast of Peninsular Malaysia. Other than an intertidal habitat preference, all other habitat preferences have independently evolved in a number of distantly related species across the tree.

**FIGURE 4:**
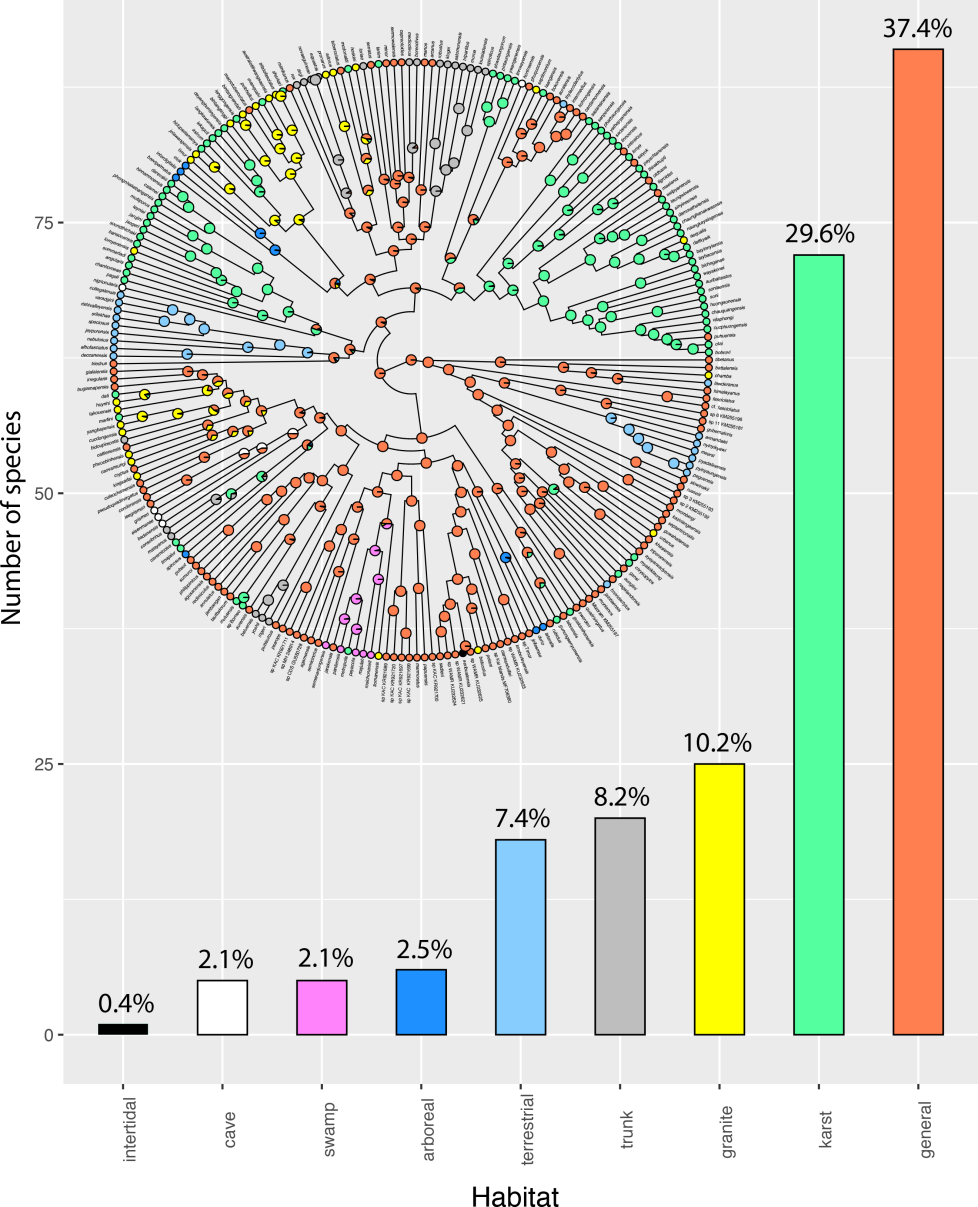
Histogram showing the percentage of species bearing each of the nine habitat preferences

These data show that for this data set, general and karst habitat preferences far out‐number that of any other habitat preference (37.4% and 29.6%, respectively vs. 0.4%–10.2%, collectively for all others; Figure 4). Of the specialized habitat preferences (i.e., non‐general), karst is at least three times more prevalent than all the others in that it occurs in 29.6% of the species followed by granite at 10.2%, trunk at 8.2%, terrestrial at 7.4%, arboreal at 2.5%, swamp and cave at 2.1%, and intertidal at 0.4% (Figure 4).

**FIGURE 5:**
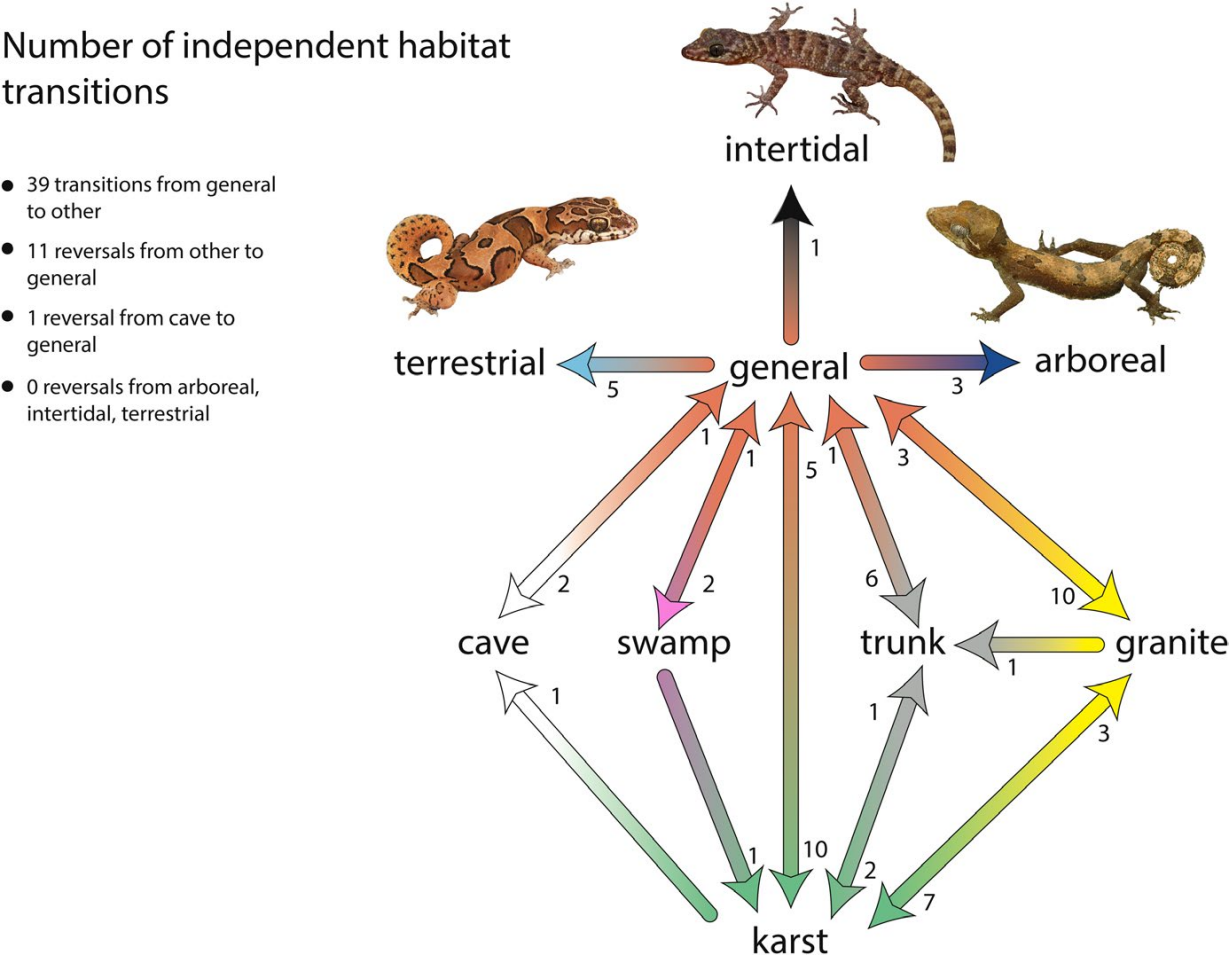
Flow chart illustrating the direction and number of habitat preference transitions recovered from the character transition matrix rounded to the nearest whole number. Values at the arrow tips represent the number of times that transition has occurred between opposing habitat preferences. All habitat transitions ultimately stem from a general habitat preference. Illustrations of three highly specialized species representing habitat preferences that did not revert to the general condition are *Cyrtodactylus srilekhae* (terrestrial), *C. seribuatensis* (interditdal), and *C. elok* (arboreal)

These results demonstrate that habitat preferences in *Cyrtodactylus* are closely associated with clades from specific geographic regions (Figure 3b). A general habitat preference is the ancestral condition in *Cyrtodactylus* which has given rise to all other habitat preferences—most of them multiple times (Figures 3b and 5). Species with a general habitat preference gave rise to intertidal species once, swamp species at least twice, arboreal and cave species at least three times, terrestrial species at least five times, trunk species at least six times, and granite and karst species at least 10 times (Figure 5). With the exception of terrestrial, arboreal, and intertidal species, some species revert back to a general habitat preference in relatively low frequencies—once for swamp, cave, and trunk species, three times for granite species, and five times for karst species (Figure 5). Interestingly, the morphologically specialized terrestrial and arboreal species (Grismer & Grismer, in prep.) and the presumably physiologically specialized intertidal species have not reverted to the ancestral condition. Additionally, karst species gave rise to cave and trunk species at least once and at least three times to granite species. Granite species gave rise to karst species at least seven times and to trunk species at least once. The higher transition frequencies between karst and granite species would indicate that transitioning between these rock types is ecomorphologically uncomplicated (Grismer et al. in prep.).

## 4 DISCUSSION

The results clearly indicate that *Cyrtodactylus* is composed of ecologically and morphologically labial scansorial (i.e., climbing) species as evidenced by the fact that various habitat preferences have evolved independently multiple times across the vast distribution of the genus from an ancestral habitat preference. Furthermore, with the exception of the morphologically specialized terrestrial and arboreal species and the physiologically specialized intertidal species, a number of habitat transitions have occurred among non‐general species as well (Figure 3). Lastly, many of these more specialized habitat preferences have reverted back to the ancestral condition a number of times independently. This ecological lability has presumably contributed to *Cyrtodactylus* being the most diverse gekkotan genus with well over 300 species. The next most diverse genus is Southeast Asian *Cnemaspis* with 57 species (Uetz et al., 2020).

### 4.1 The ecological lability of *Cyrtodactylus* and the loss of a digital adhesive system

These data demonstrate that *Cyrtodactylus* is an ecologically labile species that has frequently and independently transitioned from a general habitat preference to using other more specialized habitats. Although there may be a variety of factors that enable these transitions (Losos, 2010; Lovette et al., 2002), the potential loss of an adhesive system (i.e., toe pads) is a likely candidate. Secondary simplification of novel locomotor structures in squamates has occurred multiple times and potentially contributed to exceptional lineage divergences (Skinner et al., 2008; Wiens & Slingluff, 2001) by allowing them to locomote across a broader range of substrates as opposed to being more restricted to, and specialized for, a particular substrate. The gekkotan adhesive system is a complex, modularized system of internal and external structures (Collins et al., 2015; Higham et al., 2015) that has experienced repeated gains (11) and losses (9) within the Gekkota (Gamble et al., 2012). Not only are *Cyrtodactylus* the most diverse gekkotan lineage, they are one of nine lineages that may have secondarily lost the digital adhesive system (Russell & Gamble, 2019) which may in part, be responsible for their large, putatively adaptive radiation in a wide variety of niches and substrates from South Asia to Melanesia. The ability to locomote in specific habitats with or without toe pads is an important characteristic of geckos (Higham et al., 2015), and further research on the modular nature of foot morphology (e.g., Naylor & Higham, 2019; Russell & Gamble, 2019) will likely reveal the complex nature of its contribution to the partitioning of spatial and ecological niches.

### 4.2 Evolutionary consequences of specialization

The results of this study mirror observations from other large groups where a change in habitat preference from a generalist to something more specialized happens more frequently than the reverse (Day et al., 2016). These data suggest that species living in a generally unrestrictive ecological environment may be genetically predisposed to evolve more rapidly into new environments (Kenkel & Matz, 2016; Taute et al., 2014). These data also suggest that hypotheses positing that extreme habitat specialization such as that seen in the terrestrial and arboreal specialists or even the presumed physiological specialization as seen in the intertidal species may lead to an evolutionary “dead end” (Fernandez‐Mazuecos et al., 2013; Nosil & Mooers, 2005; Tripp & Manos, 2008; Vamosi et al., 2014). Selection pressures for novel resources in adaptive landscapes may be so strong and they prevent populations from utilizing other resources (Schluter, 2000; Wainwright & Price, 2016). However, this is not always the case, and a number of studies show that specialization does not necessarily lead to a reduction in diversity or extinction (Hardy & Otto, 2014; Johnson et al., 2009; Stireman, 2005). These mixed results, however, may be due to these studies not addressing the degrees of specialization, the method of coding characters in the analyses (i.e., discrete versus continuous, see Stephens & Wiens, 2003), or more importantly incomplete sampling (Wainwright & Price, 2016). Thus, legitimately comparable effects of specialization on macroevolutionary patterns across a broad taxonomic spectrum may not be discernable (Day et al., 2016). Each study may reveal different processes operating in a particular system or clade of unrelated species but with only limited generalizability across systems. In *Cyrtodactylus*, transitions from a general habitat preference to all others occurred at least 39 times whereas the reversal happened only 11 times and no reversals involved the highly specialized intertidal, terrestrial, and arboreal species (Figure 5). Therefore, any departure from a generalized life style in this analysis shows a tendency to limit the capacity for future evolutionary change. We are aware these numbers will change with the addition of species to the data matrix and/or detailed ecological studies that may alter character state coding for existing species but we believe the results of this study are robust enough that the generalities of our conclusions will remain unaltered.

### 4.3 Ecology and conservation of karst landscapes

Broad‐scaled studies pertaining to ecosystems management are becoming more commonplace in light of climate change and widespread habitat destruction. Such studies reconcile data from a wide range of disciplines in order to address issues that may bear on ecosystems management. Foundational to many of these studies is a basic understanding of species ecology (Cabral et al., 2009; Harfoot et al., 2014). Baseline information on habitat and microhabitat requirements of any species are key components to understanding how species interact with their environment (e.g., Grant & Grant, 2008; Greene, 2005; Losos, 2010) and as such, the contextualization of ecosystem management may ultimately turn on this simple point (Meiri, 2018; Sinervo et al., 2010).

High degrees of endemism within, and diversity among isolated karstic hills, caves, and towers result from a multitude of ecological niches afforded by their complex terrain along with their highly fragmented habitat‐island nature. Their high levels of biodiversity and site‐specific endemism rival those of most other habitats throughout the tropics, yet karstic regions are emerging as some of the most imperiled ecosystems on the planet (see discussions in Clements et al., 2006; Chung et al., 2014; Grismer, Wood, Anuar, Davis, et al., 2016; Luo et al., 2016). Indochina and Southeast Asia harbor more karst habitat than anywhere else on earth (Day & Urich, 2000; Gillieson, 2005) but unregulated and unsustainable quarrying practices continue to threaten their integrity and are the primary threat to the survival of karst‐associated species across the taxonomic spectrum. Recent studies have shown that there are far more karst‐associated species in these regions than previously suspected (e.g., Xu, 1995; Kiew, 1998, 2001, 2005; Barjadze et al., 2015; this study and Grismer, Wood, Anuar, Davis, et al., 2016; Luo et al., 2016; Connette et al., 2017; Wood et al., 2017; Ampai et al., 2019) and the rate of discovery of new species of karst‐associated amphibians and reptiles shows no signs of leveling off (see discussions in Grismer, Wood, Anuar, Davis, et al., 2016; Grismer, Wood, Anuar, Grismer, et al., 2016; Grismer, Wood, Aowphol, et al., 2017; Wood et al., 2017). This is strikingly clear for *Cyrtodactylus* in Indochina and Sundaland (Nguyen et al., 2014; Luo et al., 2016; Grismer, Wood, Thura, Zin, et al., 2018; Grismer, Wood, Thura, Win, et al., 2018; Grismer, Wood, Quah, Grismer, et al., 2020; Grismer, Wood, Quah, Thura, et al., 2019; Grismer, et al., 2020b; Davis et al., 2019; Quah et al., 2019; Wood et al., 2020). The sad irony is that despite these regions constituting some of the most extensive karstic terranes in Asia, much of it is also the least legally protected and in some areas, only 1% is recognized as vulnerable (Clements et al., 2006; Day & Urich, 2000). Therefore, the diversity of the karst‐associated species in general and *Cyrtodactylus* in particular is afforded no form of legal protection. Unfortunately, the immense financial returns from cement manufacturing make the challenge of karst conservation difficult and continued exploitation of karstic habitats for limestone shows no signs of abating.

This analysis unequivocally indicates that karstic landscapes are exceedingly important for not only maintaining *Cyrtodactylus* diversity, but serving as foci for speciation in providing the appropriate environment for the largest, independent, radiations in the genus (see Grismer, Wood, Thura, Zin, et al., 2018; Grismer, Wood, Thura, Zin, Quah, et al., 2018; Grismer, Wood, Thura, Quah, et al., 2018; Grismer, Wood, Quah, Grismer, et al., 2020a, b; Grismer, Wood, Quah, Thura, et al., 2019; Grismer et al., 2020; Figure 3b). The common reference to karstic regions as “imperiled arcs of biodiversity” is not only somewhat misleading but potentially dangerous. Karstic regions are not just refugia harboring the remnants of local biodiversity but are ecological platforms for speciation that not only *continues* to generate the most speciose, independent, radiations across the Gekkonidae but across a broad range of other taxonomic groups as well (e.g., Barjadze et al., 2015; Chen et al., 2013; Chin, 1977; Chung et al., 2014). Referring to them as “arcs of biodiversity” instead of centers for speciation devaluates their importance as generators of biodiversity in an era of biodiversity crisis and could potentially lessen the urgency for legislative conservation measures.

## CONFLICT OF INTEREST

None declared.

## AUTHOR CONTRIBUTION

**L. Lee Grismer:** Conceptualization (equal); Data curation (equal); Formal analysis; Methodology; Writing‐original draft (equal); Writing‐review & editing (equal). **Perry L. Wood, Jr.:** Writing‐review & editing (equal). **Minh Duc Le:** Data curation (equal); Writing‐review & editing (equal). **Evan S. H. Quah:** Writing‐review & editing (equal). **Jesse L. Grismer:** Conceptualization (equal); Writing‐review & editing (equal).

## Supporting information

Figure S1

Figure S2

Table S1

Supplementary Material

## Data Availability

A data table outlining the habitat preference of each species used in the analysis, the sources for coding the habitat preference for each species, GenBank numbers of the species used in the BEAST analysis, and references for habitat preferences are available within the Supporting Information.
