# Supplementary figures and images for: Evolution of habitat preference in 243 species of Bent‐toed geckos (Genus Cyrtodactylus Gray, 1827) with a discussion of karst habitat conservation
Source: Ecol Evol. 2020 Nov 22;10(24):13717–30. doi: 10.1002/ece3.6961 (PMC7771171; doi:10.1002/ece3.6961)

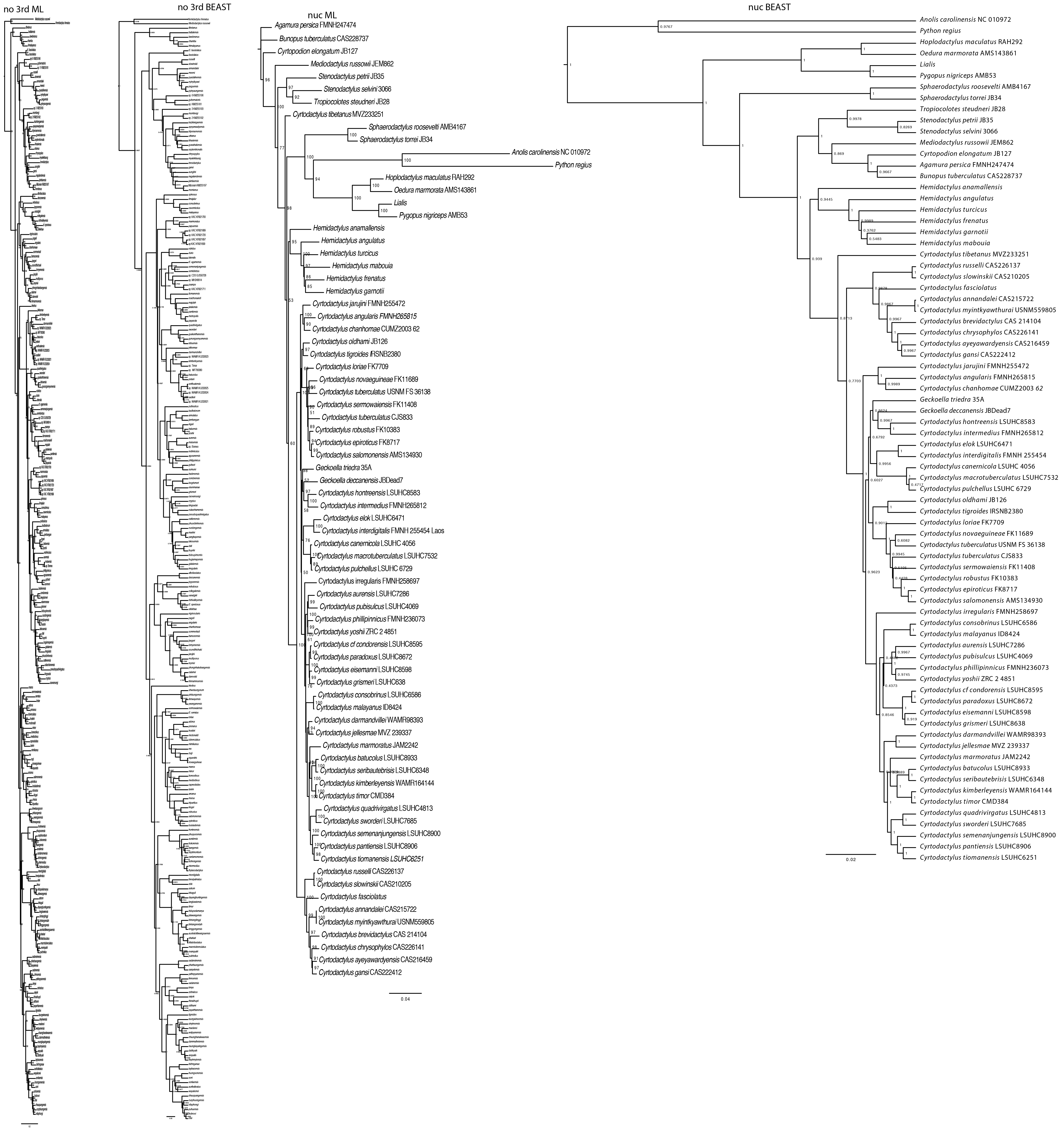

Supplement: Supplementary file 2 — Figure S2 [file ECE3-10-13717-s002.tif]
