# Supplementary material for: Evolution of habitat preference in 243 species of Bent‐toed geckos (Genus Cyrtodactylus Gray, 1827) with a discussion of karst habitat conservation
Source: Ecol Evol. 2020 Nov 22;10(24):13717–30. doi: 10.1002/ece3.6961 (PMC7771171; doi:10.1002/ece3.6961)
Supplement: Supplementary file 3 — Table S1 [file ECE3-10-13717-s003.docx]

| **Table 1S. Species, habitat preference designation with supporting references and GenBank accession numbers for specimens used in the SCM analysis.** | | | |
| --- | --- | --- | --- |
| **species** | **habitat** | **reference** | **GenBank no.** |
| *Cyrtodactylus adorus* | granite | Shea et al., (2011) | KT363927 |
| *C. aequalis* | granite | Gismer et al., (2018a) | MF872275 |
| *C. agamensis* | general | based on morphology | MH248907 |
| *C. agusanensis* | general | Welton et al., (2010a) | HQ154532 |
| *C. albofasciatus* | terrestrial | Agarwal & Karanth, (2015) | KM878625 |
| *C. angularis* | karst | Nabhitabhata & Chan-ard, (2005) | GU550717 |
| *C. annandalei* | terrestrial | Field notes at California Academy of Sciences | JX440524 |
| *C. annulatus* | general | Brown & Rico, (2009); Welton et al., (2009) | JX678807 |
| *C. arcanus* | general | Oliver, et al., (2012) | JQ820314 |
| *C. astrum* | karst | Grismer et al., (2012a) | JX519472 |
| *C. aunglini* | karst | Grimsr et al., (2018b) | MH764589 |
| *C. auralensis* | general | Murdoch et al., (2019) | KT013127 |
| *C. aurensis* | general | Grismer (2005) | JX440525 |
| *C. auribalteatus* | karst | Ellis & Pauwels, (2012); Sumontha et al., (2010) | AP018116 |
| *C. australotitiwangsaensis* | granite | Grismer et. al., (2012a) | JX519484 |
| *C. ayeyarwadyensis* | general | Mahony et al., (2009) | JX440526 |
| *C. badenensis* | cave | Nguyen et al., (2006) | MT953468 |
| *C. baluensis* | trunk | Hikida (1990) | GU366079 |
| *C. bansocensis* | karst | Luu et al., (2016) | MT953469 |
| *C. battalensis* | general | Khan, (1993) | KC151984 |
| *C. batucolus* | granite | Grismer et al., (2008) | JQ889178 |
| *C. bayinnyiensis* | karst | Grismer et al., (2018c) | MH198647 |
| *C. bichnganae* | karst | Ngo & Grismer, (2010) | MF169953 |
| *C. bidoupimontis* | trunk | Nazarov et. al., (2012) | MT953470 |
| *C. bintangrendah* | granite | Grismer et al., (2012a) | JX519487 |
| *C. bintangtinggi* | granite | Grismer et al., (2012a) | JX519494 |
| *C. borbrovi* | karst | Nguyen et al., (2015) | MT953471 |
| *C. bokorensis* | general | Murdoch et al., (2019) | KT013147 |
| *C. boreoclivus* | trunk | Oliver et al., (2011); Paul Oliver pers comm., (2019) | JQ820307 |
| *C. brevidactylus* | terrestrial | Field notes at California Academy of Sciences; Myint Kyaw Thura per comm., (2019) | JX440527 |
| *C. brevipalmatus* | arboreal | Grismer (2008, 2011) | MT953472 |
| *C. bugiamapensis* | general | Nazarov et al., (2012) | MT953473 |
| *C. calamei* | karst | Luu et al., (2016) | MT953474 |
| *C. caovansungi* | general | Orlov et al., (2007); | MF169954 |
| *C. capreoloides* | general | Oliver et al., (2012); Paul Oliver pers comm., (2019) | JQ820310 |
| *C. cardamomensis* | general | Murdoch et al., (2019) | KT013115 |
| *C. cattienensis* | general | Geissler et al., (2009) | MF169956 |
| *C. cavernicolus* | karst | Hikida (1990) | JX440528 |
| *C. chamba* | granite | Agarwal et al., (2018a) | KM255191 |
| *C. chanhomeae* | karst | Ellis & Pauwels (2012); Bauer et al., (2003) | JX440529 |
| *C. chaunghanakwaensis* | karst | Grismer et al., (2018c) | MH198644 |
| *C. chauquangensis* | karst | Quang et al., (2007) | MT953475 |
| *C. chrysopylos* | karst | Grismer et al. (2018d) | MH764601 |
| *C. collegalensis* | terrestrial | Mirza et al., (2010); Agarwal & Karanth (2015) | KX632365 |
| *C. condorensis* | granite | Grismer & Grismer, (2017) | KT013196 |
| *C. consobrinus* | trunk | Hikida, (1990); Grismer, (2011) | GU550725 |
| *C. cryptus* | general | Heidrich et al., (2007) | MT953476 |
| *C. cucdongensis* | granite | Schneider et al., (2014) | MF169959 |
| *C. cucphuongensis* | karst | Ngo & Chan, (2011) | MT953477 |
| *C. culaochamensis* | granite | Grismer et al., (2020) | KT013199 |
| *C. dammathetensis* | karst | Grismer et al., (2018a) | MF872277 |
| *C. darevskii* | karst |  | MT953478 |
| *C. darmandvillei* | general | Auffenberg, 1980 | JX440533 |
| *C. dati* | general | Ngo, (2013) | KT013104 |
| *C. dattkyaik* | karst | Grismer et al., (2020) | MN534902 |
| *C. dayangbuntingensis* | karst | Quah et al., (2019) | MN125090 |
| *C. deccanensis* | terrestrial | Agarwal & Karanth, (2015) | KM878615 |
| *C. durio* | arboreal | Grismer et. al., (2010) | KU893159 |
| *C. eisenmanae* | cave | Ngo, (2008) | JX440534 |
| *C. elok* | arboreal | Grismer, (2008, 2011); Dring, (1979) | JQ889180 |
| *C. epiroticus* | trunk | Kraus, 2008 | KT363952 |
| *C. equestris* | trunk | Oliver et al., (2016); Paul Oliver pers comm., (2019) | KT835458 |
| *C. evanquahi* | karst | Wood et al., (2020) | MN586889 |
| *C. fasciolatus* | general | Husain, (1993) | KM255184 |
| *C.* cf. *fasciolatus* | general | Ishan Agarawal pers. Comm. (2019) | KM255185 |
| *C. gansi* | general | Field notes at California Academy of Sciences | JX440537 |
| *C. gialaiensis* | general | Luu et al., (2017) | MT953479 |
| *C. grismeri* | cave | Ngo, (2008) | JX440538 |
| *C. guakanthanensis* | karst | Grismer et al., (2014a) | KU253576 |
| *C. gubaot* | general | Welton et al., (2010b) | GU550779 |
| *C. gubernatoris* | general | Annandale, (1913) | KM255204 |
| *C. gunungsenyumensis* | karst | Grismer et al., (2016a) | KU253584 |
| *C. guwahatiensis* | general | Agarwal et al., (2018) | KM255194 |
| *C. hidupselamanya* | karst | Grismer et al., (2016b) | KX011420 |
| *C. himalayanus* | general | Duda & Sahi, (1978); Ishan Agarwal pers. comm., (2019) | KM255187 |
| *C. hinnamnoensis* | karst | Luu et al., (2016) | MT953480 |
| *C. hontreensis* | cave | Ngo et al. (2008) | JX440539 |
| *C. hoskini* | granite | Shea et al., (2011); Reidel et al., (2020) | KT363931 |
| *C. huongsonensis* | karst | Luu et al., (2011) | MT953481 |
| *C. huynhi* | granite | Ngo & Bauer, (2008) | MF169963 |
| *C. ingeri* | trunk | Hikida, 1990 | BRK702 |
| *C. interdigitalis* | arboreal | Ellis & Pauwels (2012); Ubler, (1993); Nabhitabhata & Chan-ard, (2005); Luu et al., (2016) | JQ889181 |
| *C. intermedius* | general | Murdoch et al., (2019) | KT013138 |
| *C. irregularis* | general | Nazarov et al., (2008, 2012) | JX041341 |
| *C. jaegeri* | karst | Luu et al., (2014) | MT953482 |
| *C. jaintiaensis* | general | Agarwal et al., (2018b) | KM255195 |
| *C. jambangan* | general | Welton et al., (2010a) | GU366102 |
| *C. jarakensis* | general | Grismer et al., (2008) | JQ889185 |
| *C. jarujini* | karst | Ellis & Pauwells, (2012); Sumontha, (2008) | JX440541 |
| *C. jelawangensis* | granite | Grismer et al., (2014) | KJ659852 |
| *C. jellesmae* | general | Hayden et al., (2008) | JX440542 |
| *C. jeyporensis* | terrestrial | Agarwal & Karanth, (2015) | KM878616 |
| *C. kaziriangaensis* | general | Agarwal et al., (2018b) | KM255170 |
| *C. khasiensis* | general | Li, (2007) | KM255188 |
| *C. kimberleyensis* | general | Bauer & Doughty, (2012) | JX440544 |
| *C. kingsadai* | granite | Ziegler et al., (2013) | MT953483 |
| *C. klugei* | trunk | Kraus, (2008) | HQ401197 |
| *C. khorongensis* | general | Grismer et al. (2020b) | KT013124 |
| *C. laangensis* | karst | Murdoch et al., (2019) | KT013158 |
| *C. langkawiensis* | karst | Grismer et al., (2012a) | JX519502 |
| *C. lateralis* | arboreal | Harvey et al., (2016) | KU893162 |
| *C. lawderanus* | terrestrial | Ishan Agarwal pers. comm. (2019) | KM255190 |
| *C. leegrismeri* | general | Chan & Norhayati, (2010) | KT013201 |
| *C. lekaguli* | karst | Grismer et al., (2012a) | KX011425 |
| *C. lenggongensis* | karst | Grimser et al., (2016b) | JX519488 |
| *C. lenya* | karst | Connette et al., (2018) | KY041653 |
| *C. limajalur* | karst | Davis et al., (2019) | MK477177 |
| *C. linnoensis* | karst | Grismer et al., (2018a) | MF872295 |
| *C. linnwayensis* | karst | Grismer et al., (2018a) | MF872288 |
| *C. lomyenensis* | granite | Ngo & Pauwels, (2010) | MF169966 |
| *C. loriae* | trunk | dan Mulyadi, (2007); Oliver et al., (2011) | JQ820299 |
| *C. louisiadensis* | trunk | Kraus, (2008); Harlow, (2013) | HQ401190 |
| *C. macrotuberculatus* | general | Grismer & Norhayti, (2008) | JX519519 |
| *C. maelanoi* | general | Grismer et al., (2020c) | MT823267 |
| *C. majulah* | swamp | Grismer et al. (2012b) | JX988529 |
| *C. malayanus* | trunk | Hikida, (1990); Grismer, (2011) | GU550733 |
| *C. manos* | general | Oliver et al., (2012) | JQ820322 |
| *C. marmoratus* | general | dan Mulyadi, (2007) | JX440546 |
| *C. mcdonaldi* | karst | Shea et al., (2011) | HQ401139 |
| *C. medioclivus* | general | Oliver et al., (2012); Paul Oliver pers comm., (2019) | JQ820294 |
| *C. meersi* | terrestrial | Grismer et al., (2018d) | MH624104 |
| *C. metropolis* | karst | Grismer et al., (2014b) | KU253578 |
| *C. mimikanus* | general | Brown & Parker, (1973); Paul Oliver pers. comm., (2019) | JQ820316 |
| *C. minor* | general | Oliver et al., (2012) | JQ820318 |
| *C.* Mizoram | general | Agarwal pers. comm., (2019) | KM255197 |
| *C. mombergi* | general | Grismer et al., (2019a) | MN059869 |
| *C. montanus* | general | Agarwal et al., (2018b) | KM255200 |
| *C. multiporus* | karst | Nazarov et al., (2014) | MT953484 |
| *C. muluensis* | karst | Davis et al., (2019) | MK477167 |
| *C. murua* | trunk | Kraus & Allison, (2006) | KT363953 |
| *C. myaleiktaung* | karst | Grismer et al., (2018b) | MH764589 |
| *C. nagalandensis* | general | Agarwal et al., (2018b) | KM255199 |
| *C. naungkayaingensis* | karst | Grismer et al., (2018c) | MH198664 |
| *C. nebulosus* | terrestrial | Agarwal & Karanth, (2015) | KM878618 |
| *C. nigriocularis* | cave | Nguyen et al., (2006) | MT953485 |
| *C. novaeguineae* | trunk | Oliver et al., (2011) | KT363956 |
| *C. nyinyikyawi* | terrestrial | Grismer et al., (2019b) | MH624118 |
| *C. oldhami* | general | Nabhitabhata & Chan-ard, (2005) | MF872302 |
| *C. otai* | karst | Nguyen et al. (2015) | MT953486 |
| *C. pageli* | karst | Schneider et al., (2011) | MT953487 |
| *C. pantiensis* | swamp | Grismer et al., (2008) | JQ889185 |
| *C. papuensis* | general | Brown & Parker, (1973) | JQ820320 |
| *C. payacola* | swamp | Johnson et al., (2012) | JQ889190 |
| *C. payarhtanensis* | karst | Connette et al., (2018) | KY041654 |
| *C. peguensis* | terrestrial | Grismer et al., (2018d, 2019b) | MH756190 |
| *C. petani* | general | Riyanto et al., (2015a) | KU232620 |
| *C. pharbaungensis* | karst | Grimser et al., (2018a) | MF872303 |
| *C. philippinicus* | general | Brown et al., (2009); Welton et al., (2010b) | GU550825 |
| *C. phongnhakebangensis* | karst | Loos et al., (2012) | MF169970 |
| *C. phuocbinhensis* | granite | Nguyen et al., (2013) | MT953488 |
| *C. phuquocensis* | general | Ngo et al., 2010 | MF169971 |
| *C. pinlaungensis* | karst | Grismer et al., (2019c) | MN030634 |
| *C. pronarus* | granite | Shea et al., (2011) | HQ401152 |
| *C. psarops* | general | Harvey et al., (2015) | KR921705 |
| *C. pseudoquadrivirgatus* | general | Rösler et al., (2008); Luu et al., (2016) | MF169972 |
| *C. pubisulcus* | general | Hikida, (1990) | JX440551 |
| *C. puhuensis* | general | Nguyen et al., (2014) | MT953489 |
| *C. pulchellus* | granite | Grismer et al., (2012) | MF169974 |
| *C. pyadalinensis* | terrestrial | Grismer et al., (2019b) | MH624105 |
| *C. pyinyaungensis* | terrestrial | Grismer et al. (2018a) | MF872307 |
| *C. quadrivirgatus* | general | Johnson et al., (2012); Grismer, (2012) | MF169975 |
| *C. redimiculus* | general | Brown & Dimalibot, (2009) | GU550738 |
| *C. rex* | trunk | Oliver et al., (2016); Paul Oliver pers comm., (2019) | KT835460 |
| *C. rishivalleyensis* | terrestrial | Agarwal, (2016) | KX698081 |
| *C. robustus* | trunk | Kraus, (2008); Paul Oliver pers comm., (2019) | JX440554 |
| *C. rosichonariefi* | swamp | Riyanto et al. (2015b) | KP256188 |
| *C. rubidus* | general | Annandale (1913); Chnadramouli (2020) | KM255203 |
| *C. russelli* | general | Grismer et al. (2019) | JX440555 |
| *C. sadanensis* | karst | Grismer et al. 2018a | MF872324 |
| *C. sadansinensis* | karst | Grismer et al. (2018a) | MF872325 |
| *C. sadleiri* | general | Smith et al., (2012) | MH105038 |
| *C. saiyok* | general | Aksornneam 2019: abstract; Panitvong et al. 2014 | MF872308 |
| *C. salomonensis* | trunk | Rösler et al., (2007) | JX440556 |
| *C. sanpelensis* | karst | Grismer et al., (2018a) | MF872345 |
| *C. semenanjungensis* | swamp | Grismer & Leong, (2005) | JQ889177 |
| *C. semicinctus* | general | Harvey et al., (2015) | KR921713 |
| *C. septentrionalis* | general | Agarwal et al., (2018b) | MH971164 |
| *C. septimontium* | granite | Murdoch et al., (2019) | MH940233 |
| *C. seribuatensis* | intertidal | Youmans & Grismer, (2006) | JX440557 |
| *C. sermowaiensis* | general | Rösler et al., (2007) | JQ820296 |
| *C. serratus* | trunk | Kraus, (2007); Paul Oliver pers comm., (2019) | JQ820297 |
| *C. sharkari* | karst | Grismer et al., (2014c) | KJ659853 |
| *C. shwetaungorum* | karst | Grismer et al., (2018a) | MF872353 |
| *C. sinyineensis* | karst | Grismer et al., (2018a) | MF872355 |
| *C. slowinskii* | general | Field notes at California Academy of Sciences | JX440559 |
| *C. sommerladi* | karst | Luu et al., (2016) | MT953490 |
| *C. soni* | karst | Le et al., (2016) | MT953491 |
| *C. sonlaensis* | karst | Nguyen et al., (2017) | MT953492 |
| *C. soundthichaki* | karst | Luu et al., (2015) | MT953493 |
| *C. speciosus* | terrestrial | Mirza et al., (2010); Agarwal & Karanth, (2015) | KM878629 |
| *C. spinosus* | arboreal | Linkem et al., (2008) | MT953494 |
| *C. srilekhae* | terrestrial | Agarwal, (2016) | KX698084 |
| *C. sumuroi* | general | Welton et al., (2010a) | GU550772 |
| *C. sworderi* | general | Grismer, (2011) | JQ889189 |
| *C. takouensis* | granite | Ngo & Bauer, (2008) | MF169978 |
| *C. tanim* | karst | Nielson & Oliver, (2017) | MF706378 |
| *C. taungwineensis* | karst | Grismer et al. (2020) | MN534925 |
| *C. tautbatorum* | general | Welton et al., (2009) | GU550752 |
| *C. taybacensis* | karst | Pham et al., (2019) | MT953495 |
| *C. tebuensis* | general | Grismer et al., (2013) | JX988528 |
| *C. teyniei* | karst | David et al., (2011) | MT953496 |
| *C. thirakhupti* | karst | Pauwels et al., (2004); pers. obs. | AP018115 |
| *C. thylacodactylus* | terrestrial | Murdoch et al., (2019); pers. obs. | KT013163 |
| *C. tibetanus* | general | Shi & Zhoa, (2010); Kai Wang, pers. comm., (2019) | JX440561 |
| *C. tigroides* | karst | Bauer et al., (2003); Aksornneam, (2019) | JX440562 |
| *C. timur* | granite | Grismer et al., (2016) | KJ659857 |
| *C. tiomanensis* | granite | J. Grismer et al., (2004) | GU550734 |
| *C. triedrus* | general | Agarwal & Karanth, (2015); Botejue et al., (2012) | JX440522 |
| *C. trilatofasciatus* | granite | Grismer et al., (2012a) | JX519530 |
| *C. tripartitus* | trunk | Kraus, (2008) | JQ820317 |
| *C. triperanensis* | general | Ishan Agarwal pers. comm., (2019) | KM255183 |
| *C. tuberculatus* | general | Shea et al., (2011); Worthington & Couper, (2015) | KT363943 |
| *C. urbanus* | granite |  | MN911174 |
| *C. varadgirii* | terrestrial | Agarwal, (2016) | KX632368 |
| *C. vilaphongi* | karst | Schneider et. al., (2014); Luu et al., (2016) | MT953497 |
| *C. wayakonei* | karst | Nguyen et al., (2010); Yuan & Rao, (2011); Luu et al., (2016) | MT953498 |
| *C. welpyanensis* | karst | Grismer et al. (2018d) | MF872360 |
| *C. yangbayensis* | granite | Ngo & Chan, (2010) | KT013202 |
| *C.* cf. *yangbayensis* | karst | Ngo, (2011) | MF169968 |
| *C. yathepyanensis* | karst | Grismer et al., (2018a) | MF872363 |
| *C. yoshii* | trunk | Hikida, (1990) | JX440565 |
| *C. ywanganensis* | karst | Grismer et al., (2018a) | MH607610 |
| *C. zebriacus* | general | Taylor, (1963); P. L. Wood, pers. comm. (2019) | MF100157 |
| *C. zugi* | trunk | Oliver et al., (2008) | JQ820306 |
| *Cyrtodactylus* sp. KM255181 | general | Ishan Agarwal pers. comm., (2019) | KM255181 |
| *Cyrtodactylus* sp. KAC KR921689 | general | Kyle O'Connell pers. comm., (2020) | KR921689 |
| *Cyrtodactylus* sp. KR921697 | general | Kyle O'Connell pers. comm., (2020) | KR921697 |
| *Cyrtodactylus* sp. KR921699 | general | Kyle O'Connell pers. comm., (2020) | KR921699 |
| *Cyrtodactylus* sp. KR921700 | general | Kyle O'Connell pers. comm., (2020) | KR921700 |
| *Cyrtodactylus* sp. KR921711 | general | Kyle O'Connell pers. comm., (2020) | KR921711 |
| *Cyrtodactylus* sp. KR921720 | general | Kyle O'Connell pers. comm., (2020) | KR921720 |
| *Cyrtodactylus* sp. MF706380 | general | Nielson & Oliver, (2017) | MF706380 |
| *Cyrtodactylus* sp. Timor | general | Nielson & Oliver, (2017) | JX440560 |
| *Cyrtodactylus* sp. WAMR KU232623 | general | Riyanto et al., (2020) | KU232623 |
| *Cyrtodactylus* sp. 2 KM255192 | general | Ishan Agarwal pers. comm., (2019) | KM255192 |
| *Cyrtodactylus* sp. WAMR KU232625 | general | Riyanto et al., (2020) | KU232625 |
| *Cyrtodactylus* sp. WAMR KU232621 | general | Riyanto et al., (2020) | KU232621 |
| *Cyrtodactylus* sp. WAMR KU232624 | general | Riyanto et al., (2020) | KU232624 |
| *Cyrtodactylus* sp. MH248914 | general | Kyle O'Connell pers. comm., (2020) | MH248914 |
| *Cyrtodactylus* sp. 3 KM255193 | general | Ishan Agarwal pers. comm., (2019) | KM255193 |
| *Cyrtodactylus* sp. 6 KM255196 | general | Ishan Agarwal pers. comm., (2019) | KM255196 |
| *Cyrtodactylus* sp. Borneo | karst | Oliver et al., (2012) | MF706373 |
| *Cyrtodactylus* sp. CDS GU550728 | general | inferred from the phylogeny (Fig. xx) | GU550728 |
