# Supplementary material for: Evolution of habitat preference in 243 species of Bent‐toed geckos (Genus Cyrtodactylus Gray, 1827) with a discussion of karst habitat conservation
Source: Ecol Evol. 2020 Nov 22;10(24):13717–30. doi: 10.1002/ece3.6961 (PMC7771171; doi:10.1002/ece3.6961)
Supplement: Supplementary file 4 — Supplementary Material [file ECE3-10-13717-s004.docx]

Agarwal, I. (2016). Two new species of ground-dwelling *Cyrtodactylus* (*Geckoella*) from the Mysore Plateau, south India. *Zootaxa*, 4193, 228–244.

Agarwal, I., Karanth, K. P. (2014). A phylogeny of the only ground-dwelling radiation of *Cyrtodactylus* (Squamata, Gekkonidae): diversification of *Geckoella* across peninsular India and Sri Lanka. *Molecular Phylogenetics and Evolution*, 82, 193–199.

Agarwal, I. Khandekar, A., Bauer, A. M. (2018b). A new bent-toed gecko (Squamata: Gekkonidae) from the Western Himalayas, Himachal Pradesh, India *Zootaxa*, 4446, 442–454.

Agarwal, I., Stephen., Varad, B., Giri, R., Chaitanya, Bauer, A. B. (2018a). Six new *Cyrtodactylus* (Squamata: Gekkonidae) from northeast India. Zootaxa, 4524, 501–535.

Annandale, N. (1913). The Indian geckos of the genus *Gymnodactylus*. *Records of the Indian Museum*, 9, 309­­–326.

Auffenberg, W. (1980). The herpetofauna of Komodo, with notes on adjacent areas. *Bulletin of the Florida State Museum of Natural History*, *Biological Sciences, 25*, 39–156.

Bauer, A. M. & Doughty, P. (2012). A new bent-toed gecko (Squamata: Gekkonidae: *Cyrtodactylus*) from the Kimberley region, Western Australia. *Zootaxa*, 3187, 32–42.

Botejue, W., Madhava S., Wattavidanage, J. (2012). Herpetofaunal diversity and distribution in Kalugala proposed forest reserve, Western province of Sri Lanka. *Amphibian & Reptile Conservation*, 5, 65–80.

Brown, R., & Dimalibot, J. (2009). *Cyrtodactylus redimiculus.* The IUCN Red List of Threatened Species. Version 2014.2 Available at: www.iucnredlist.org (accessed on 14 October 2014).

Brown, W.C., & Parker, F. (1973). A new species of *Cyrtodactylus* (Gekkonidae) from New Guinea with a key to species from the island. *Brevoria*, 417, 1–7.

Brown, R., & Rico, E. (2009). *Cyrtodactylus annulatus.* The IUCN Red List of Threatened Species. Version 2014.2 Available at: www.iucnredlist.org (accessed on 14 October 2014).

Brown, R., Gaulke, M., & Rico, E. (2009). *Cyrtodactylus philippinicus*. The IUCN Red List of Threatened Species. Version 2014.2 Available at: www.iucnredlist.org (accessed on 14 October 2014).

Chan, K. O. & Norhayati, A. (2005). A new insular species of *Cyrtodactylus* (Squamata: Gekkonidae) from northeastern Peninsula Malaysia, Malaysia. *Zootaxa*, 2389, 47–56.

Chandramouli, S.R. (2020). A review of the gekkonid genus *Cyrtodactylus* Gray, 1827 (Sauria: Gekkonidae) in the Andaman and Nicobar archipelago with the description of two new species from the Nicobar Islands. *Asian Journal of Conservation Biology,* 9, 78–89.

Connette, G. M., Oswald, P., Thura, M. K., Connette, KJ, Grindley, M. E., Songer, M., et al. (2017). Rapid forest clearing in a Myanmar proposed national park threatens two newly discovered species of geckos (Gekkonidae: *Cyrtodactylus*). *PLoS One* 12, 4, e0174432

dan Mulyadi, A. H. (2007). Herpetofauna of Waigeo Island (Herpetofauna di Pulau Waigeo). *Zoological Museum of Bogoriense, Indonesia.* Unpublished.

David, P., Nguyen, T. Q., Schneider, N., & Ziegler, T. (2011). A new species of *Cyrtodactylus* Gray, 1827 from central Laos (Squamata: Gekkonidae). *Zootaxa*, 2833, 29–40.

Davis, H. R., Bauer, A. M., Jackman, T. R., Nashriq, I., & Das, I. (2019). Uncovering karst endemism within Borneo: two new *Cyrtodactylus* species from Sarawak, Malaysia. *Zootaxa,* 4614, 331–352.

Dring, J. C. M. (1979). Amphibians and reptiles from northern Trengganu, Malaysia, with descriptions of two new geckos: *Cnemaspis* and *Cyrtodactylus*. *Bulletin of the British Museum (Natural History)*, 34, 181–241.

Duda, P. L., & Sahi, D. N. (1978). *Cyrtodactylus himalayanus*: A new gekkonid species from Jemmu, India. *Journal of Herpetology*, 12, 351–354.

Ellis, M., & Pauwels, O. S. G. (2010). The bent-toed geckos (*Cyrtodactylus*) of the caves and karst of Thailand. *Cave and Karst Science*, 39, 16–22.

Geissler, P., Nazarov, R., Orlov, N. L., Bohme, W., Phung, T. M., Nguyen, T. Q., & Ziegler, T. (2009). A new species of the *Cyrtodactylus irregularis* complex (Squamata: Gekkonidae) from southern Vietnam. *Zootaxa*, 2161, 20–32.

Grismer, J. L., Grismer, L. L., Das, I., Yaakob, N. S., Lim, L. B., Tzi, M. L., Youmans, T. M., & Kaiser, H. (2004). Species diversity and checklist of the herpetofauna of Pulau Tioman, Peninsular Malaysia, with a preliminary overview of habitat utilization. *Asiatic Herpetological Research*, 10, 247–279.

Grismer, L. L. (2005) New species of bent-toed gecko (*Cyrtodactylus* Gray 1827) from Pulau Aur, Johor, West Malaysia. *Journal of Herpetology*, 39, 424–432.

Grismer, L. L. (2008) On the distribution and identification of *Cyrtodactylus brevipalmatus* Smith, 1923, and *Cyrtodactylus elok*, Dring, 1979. *The Raffles Bulletin of Zoology*, 56, 177–179.

Grismer, L.L. (2011) *Lizards of Peninsular Malaysia, Singapore and Their Adjacent Archipelagos*. Edition Chimaira, Frankfürt am Main, 728 pp.

Grismer, L. L., Anuar, S., Quah, E. S., Muin, M. A., Chan, K. O., Grismer, J. L., & Norhayati, A. (2010). A new spiny, prehensile-tailed species of *Cyrtodactylus* (Squamata: Gekkonidae) from Peninsular Malaysia with a preliminary hypothesis of relationships based on morphology. *Zootaxa*, 2625, 40–52.

Grismer, L. L., Anuar, S., Muin, M. A., Quah, E. S., & Wood, P. L., Jr. (2013). Phylogenetic relationships and description of a new upland species of Bent-toed Gecko (*Cyrtodactylus* Gray, 1827) of the *C. sworderi* complex from northeastern Peninsular Malaysia. *Zootaxa*, 3616, 239–52.

Grismer, L. L., Belabut, D. M., Quah, E. S. H. Q., Chan, K. O., Wood, P. L., Jr., & Hasim, R. (2014a). A new species of karst forest-adapted Bent-toed Gecko (genus *Cyrtodactylus* Gray, 1827) belonging to the *C. sworderi* complex from a threatened karst forest in Perak, Peninsular Malaysia. *Zootaxa*, 3755, 434–446.

Grismer, L. L., Chan, K. O., Grismer, J. L., Wood, Jr, P. L., & Belabut, D. M. (2008). Three new species of *Cyrtodactylus* (Squamata: Gekkondiae) from Peninsular Malaysia. *Zootaxa*, 1921, 1–23.

Grismer, L.L., Chan, K.O., Oaks, J.R., Neang, T., Lang, S., Murdoch, M.L., Stuart, B.L. & Grismer J.L. (2020b). A new insular species of the *Cyrtodactylus intermedius* (Squamata: Gekkonidae) group from Cambodia with a discussion on habitat preference and morphology. Zootaxa, in press.

Grismer, L. L., & Grismer, J. L. (2017). A re-evaluation of the phylogenetic relationships of the *Cyrtodactylus condorensis* group (Squamata; Gekkonidae) and a suggested protocol for the characterization of rock-dwelling ecomorphology in *Cyrtodactylus*. *Zootaxa*, 4300, 486–504.

Grismer, L. L., & Leong, T. M. (2005). New species of *Cyrtodactylus* (Squamata: Gekkonidae) from Southern Peninsular Malaysia. *Journal of Herpetology*, 39, 584–591.

Grismer, L. L. & Norhayati, A. (2008). A new insular species of *Cyrtodactylus* (Squamata: Gekkonidae) from the Langkawi Archipelago, Kedah, Peninsular Malaysia. *Zootaxa*, 1924, 53–68.

Grismer, L.L., Rujirawan, A., Termprayoon, K., Ampai, N., Yodthong, S., Wood, Jr., P.P., & Aowphol, A. (2020c). A new species of *Cyrtodactylus* Gray (Squamata; Gekkonidae) from the Thai Highlands with a discussion on the evolution of habitat preference. *Zootaxa,* in press.

Grismer, L. L., Wood, Jr., P. L., Anuar, S., Davis, H. R., Cobos, A. J., & Murdoch, M. L. (2016a). A new species of karst forest Bent-toed Gecko (genus *Cyrtodactylus* Gray) not yet threatened by foreign cement companies and a summary of Peninsular Malaysia’s endemic karst forest herpetofauna and the need for its conservation. *Zootaxa,* 4061, 1–17.

Grismer, L.L.; Wood, J., P. L., Anuar, S., Grismer, M. S., Quah, E. S. H., Murdoch, M. L., Muin, M. A., Davis, H. R., Aguliar, C., Klabacka, R., Cobos, A. J., Aowphol, A., Sites, Jr., J. R. (2016b). Two new Bent-toed Geckos of the *Cyrtodactylus pulchellus* complex from Peninsular Malaysia and multiple instances of convergent adaptation to limestone forest ecosystems. *Zootaxa*, 4105, 401–429.

Grismer, L. L., Wood, Jr., P. L., Anuar, S.,, Quah, E. S. H., Muin, M. A., Maketab, M., Chan, K. O., Sumarli, A. X., Loredo, A. I., & Heinz, H. M. (2014c). The phylogenetic relationships of three new species of the *Cyrtodactylus pulchellus* complex (Squamata: Gekkonidae) from poorly explored regions in northeastern Peninsular Malaysia. *Zootaxa,* 3786, 359–381.

Grismer, L. L., Wood, P. L., Jr., Chan, K. O.., Anuar, S., & Muin, M.A. (2014b). Cyrts in the city: a new Bent-toed Gecko (Genus *Cyrtodactylus*) is the only endemic species of vertebrate from Batu Caves, Selangor, Peninsular Malaysia. *Zootaxa*, 3774, 381–394.

Grismer, L. L., Wood Jr, P. L., & Lim, K.K .P. (2012b). *Cyrtodactylus majulah*, a new species of bent-toed gecko (Reptilia: Squamata: Gekkonidae) from Singapore and the Riau Archipelago. *The Raffles Bulletin of Zoology*, 60, 487–499.

Grismer, L. L., Wood, Jr., P. L., Myint Kyaw Thura, Nay Myo Win, Grismer, M. S., Trueblood, T. A., & Quah, E. S. H. (2018b). A re-description of *Cyrtodactylus chrysopylos* Bauer (Squamata: Gekkonidae) with comments on the adaptive significance of orange coloration in hatchlings and descriptions of two new species from eastern Myanmar (Burma). *Zootaxa*, 4527, 151–185.

Grismer, L. L., Wood, Jr., P. L., Myint Kyaw Thura, Nay Myo Win, Quah, E. S. H. (2019b). Two more new species of the *Cyrtodactylus peguensis* group (Squamata: Gekkonidae) from the fringes of the Ayeyarwady Basin, Myanmar. *Zootaxa*, 4577, 274–294.

Grismer, L. L., Wood Jr., P. L., Myint Kyaw Thura, Quah, E. S. H., Grismer, M. S., Murdoch, M. L., Espinoza, R. E., & Aung Lin. (2018d). A new *Cyrtodactylus* Gray, 1827 (Squamata, Gekkonidae) from the Shan Hills and the biogeography of Bent-toed Geckos from eastern Myanmar. *Zootaxa,* 4446, 477–500.

Grismer, L. L., Wood Jr., P. L., Myint Kyaw Thura, Quah, E. S. H., Murdoch, M. L., Grismer, M. S., Herr, M. W., Aung Lin, & Htet Kyaw. (2018c). Three more new species of *Cyrtodactylus* (Squamata: Gekkonidae) from the Salween Basin of eastern Myanmar underscore the urgent need for the conservation of karst habitats. *Journal of Natural History*, 52, 1243–1294.

Grismer, L. L., Wood, Jr., P. L., Myint Kyaw Thura, Thaw Zin, Quah, E. H. S., Murdoch, M. L., Grismer, M. S., Aung Lin, Htet Kyaw, & Ngwe Lwin (2018a). Twelve new species of *Cyrtodactylus* Gray (Squamata: Gekkonidae) from isolated limestone habitats in east- central and southern Myanmar demonstrate high localized diversity and unprecedented microendemism *Zoological Journal of the Linnean Society,* 182, 862–959.

Grismer, L. L., Wood, Jr, P. L., Quah, E. S. H., Anuar, S., Muin, M. A., Sumontha, M., Norhayati, A., Bauer, A. M., Wangkulalngkul, S., Grismer, J. L., & Pauwels, O. S. G. (2012a). A phylogeny and taxonomy of the Thai-Malay Peninsula Bent-toed geckos of the *Cyrtodactylus pulchellus* complex (Squamata: Gekkonidae): combined morphological and molecular analyses with descriptions of seven new species. *Zootaxa*, 3520, 1–55.

Grismer, L. L., Wood, Jr., P. L., Quah, E. S. H., Grismer, M. S., Thura, M. K., Oaks, J. R., Lin, A. (2020a) Two new species of *Cyrtodactylus* Gray, 1827 (Squamata: Gekkonidae) from a karstic archipelago in the Salween Basin of southern Myanmar (Burma). *Zootaxa,* 4718, 151–183.

Grismer, L. L., Wood, P. L., Jr, Quah, E. S. H., Murdoch, M. L., Grismer, M. S., Herr, M. W., Espinoza, R. E., Brown, R. M., Aung Lin. (2018d). A phylogenetic taxonomy of the *Cyrtodactylus peguensis* group (Reptilia: Squamata: Gekkonidae) with descriptions of two new species from Myanmar. *PeerJ*, 6, e5575.

Grismer, L. L., Wood, Jr., P. L., Quah, E. S. H., Myint Kyaw Thura, Herr, M. W., & Aung Ko Lin. (2019a). A new species of forest-dwelling *Cyrtodactylus* Gray (Squamata: Gekkonidae) from the Indawgyi Wildlife Sanctuary, Kachin State, Myanmar. *Zootaxa*, 4623, 1–25.

Grismer, L. L., Wood, Jr., P. L., Quah, E. S. H. Myint Kyaw Thura, Oaks, J. R., & Aung Lin. (2019c). A new species of Bent-toed Gecko (Squamata, Gekkonidae, *Cyrtodactylus*) from the Shan Plateau in eastern Myanmar (Burma). *Zootaxa*, 4624, 301–321.

Harlow, P. (2013) *Cyrtodactylus louisiadensis*. The IUCN Red List of Threatened Species. Version 2014.2 Available at: www.iucnredlist.org (accessed on 14 October 2014).

Harvey, M. B, O’connell, K. A., Barraza, G., Riyanto, A., Kurniawan, N., & Smith, E. N. (2015). Two new species of *Cyrtodactylus* (Squamata: Gekkonidae) from the Southern Bukit Barisan Range of Sumatra and an estimation of their phylogeny *Zootaxa*, 4020, 495–516.

Harvey, M. B., O’connell, K. A., Wostl, E., Riyanto, A., Kurniawan, N., Smith, E. N., Grismer, L. L. (2016). Redescription *Cyrtodactylus lateralis* (Werner) (Squamata: Gekkonidae) and Phylogeny of the Prehensile-tailed *Cyrtodactylus*. *Zootaxa*, 4107, 517–540.

Hayden, C.J., Brown, R.M., Gillespie, G., Setiadi, M.I., Linkem, C.W., Iskandar, D.T., Umilaela, Bickford, D.P., Riyanto, A., Mumpuni & McGuire, J.A. (2008). A new species of bent-toed gecko *Cyrtodactylus* Gray, 1827, (Squamata: Gekkonidae) from the island of Sulawesi, Indonesia. *Herpetologica*, 64, 109-120.

Heidrich, A., Rösler, H., Than, V.N., Böhme, W. &Ziegler, T. (2007). Another new *Cyrtodactylus* (Squamta: Gekkonidae) from Phing Nha-Ke Bang National Park, central Truoung Son, Vietnam. *Zootaxa*, 1445, 35–38.

Hikida, T. (1990). Bornean gekkonid lizards of the genus *Cyrtodactylus* (Lacertilia: Gekkonidae) with descriptions of three new species. *Japanese Journal of Herptelogy*, 13, 91–107.

Husain, A. & Ray, P. (1993). First record of *Cyrtodactylus fasciolatus* (Blyth), the bent-toed banded gecko (Sauria: Gekkonidae: Gekkoninae) from Garwhal Hills. *Journal of the Bombay Natural History Society*, 90, 518.

Johnson, C.B., Quah, E.S., Anuar, S., Muin, M.A., Wood Jr, P.L., Grismer, J.L., Greer, L.F., Chan, K.O., Norhayati, A., Bauer, A.M. & Grismer, L.L. (2012). Phylogeography, geographic variation and taxonomy of the bent-toed gecko *Cyrtodactylus quadrivirgatus* Taylor, 1962 from Peninsula Malaysia with the description of a new swamp dwelling species. *Zootaxa*, 3406, 39–58.

Khan, M. S. (1993). A new angular-toed gecko from Pakistan, with remarks on the taxonomy and a key to the species belonging to genus *Cyrtodactylus* (Reptilia: Sauria: Gekkonidae). Pakistan Journal of Zoology 25 (1): 67-73.

Kraus, F. & Allison, A. (2006). A new species of *Cyrtodactylus* (Lacertilia: Gekkonidae) from Papua New Guinea. *Zootaxa*, 1247, 59–68.

Kraus, F. (2007). A new species of *Cyrtodactylus* (Squamata: Gekkonidae) from western Papua New Guinea. *Zootaxa*, 1425, 63–68.

Kraus, F. (2008). Taxonimic partitioning of *Cyrtodactylus louisiadensis* (Lacertilia: Gekkonidae) from Papua New Guinea. *Zootaxa*, 1883, 1-27.

Li, P.-P. (2007). Description of a new subspecies of *Cyrtodactylus khasiensis* from China. *Acta Zootaxonomica Sinica*, 32, 733–737.

Linkem, C. W., McGuire, J. A., Hayden, C. J., Setiadi, M. I., Bickford, D. P., & Brown, R. M. (2008). A new species of bent-toed gecko (Gekkonidae: *Cyrtodactylus*) from Sulawesi Island, Indonesia. *Herpetologica*, 64, 224–234.

Loos, J., von Wehdren, H., Kien, N. D. & Ziegler, T. (2012). Niche segregation in microhabitat use of three sympatric *Cyrtodactylus* in the Phong Nha-Ke Bang National Park, central Vietnam. *Herpetological Conservation and Biology*, 7, 101–108.

Nahbitabhata, J. & Chan-ard, T. (2005) *Thailand Red Data: Mammals, Reptiles and Amphibians.* ONEP: Office of Natural Resources and Envrionmental Policy and Planning, Thailand.

Nazarov, R. A., Orlov, N. L., Nguyen, N. C., & Ho, T. C. (2008). Taxonomy of naked-toes geckos *Cyrtodactylus irregularis* complex of south Vietnam and description of a new species from Chu Yang Sin Natural Park (Krong Bong district, Dac Lac province, Vietnam). *Russian Journal of Herpetology*, 15, 141–156.

Nazarov, R., Poyarkov, N. A., Orlov, N. L., Phung, T. M., Nguyen, T. T., Hoang, D. M. & Ziegler, T. (2012). Two new cryptic species of the *Cyrtodactylus irregularis* complex (Squamata: Gekkonidae) from southern Vietnam. *Zootaxa*, 3302, 1–24.

Nazarov, R., Poyarkov, N. A., Orlov, Ngyuen, N.S., Mito, K.D., Martynov, A.A., Konstantinov, E.L., & Chulisov, A.S. (2014). A review of genus *Cyrtodactylus* (Reptilia: Sauria: Gekkonidae) in fauna of Laos with description of four new species. *Proceedings of the Zoological Institute RAS,* 318, 391–423.

Ngo, V. T. (2008). Two new cave-dwelling species of *Cyrtodactylus* Gray (Squamata: Gekkonidae) from Southwestern Vietnam. *Zootaxa*, 1909, 37­–51.

Ngo, V. T. (2011). *Cyrtodactylus martini*, another new karst-dwelling *Cyrtodactylus* Gray, 1827 (Squamata: Gekkonidae) from Northwestern Vietnam. *Zootaxa*, 2834, 33–46.

Ngo, V. T. (2013). *Cyrtodactylus dati*, a new forest dwelling Bent-toed Gecko (Squamata: Gekkonidae) from southern Vietnam. *Zootaxa*, 3616, 151–64.

Ngo, V. T. & Bauer, A. M. (2008). Descriptions of two new species of *Cyrtodactylus* Gray, 1827 (Squamata: Gekkonidae) endemic to southern Vietnam. *Zootaxa*, 1715, 27–42.

Ngo, V. T. & Chan, K. O. (2010). A new species of *Cyrtodactylus* Gray, 1826 (Squamata: gekkonidae) from Khanh Hoa province, Southern Vietnam. *Zootaxa*, 2504, 47–60.

Ngo, V. T. & Chan, K. O. (2011). A new karstic cave-dwelling *Cyrtodactylus* Gray (Squamata: Gekkonidae) from Northern Vietnam. *Zootaxa*, 3125, 51–63.

Ngo, V. T. & Grismer, L. L. (2010). A new karst dwelling *Cyrtodactylus* (Squamata: Gekkonidae) from Son La province, north-western Vietnam. *Hamadryad*, 35, 84–95.

Ngo, V.T., Grismer, L. L. & Grismer, J. L. (2008). A new endemic cave dwelling species of *Cyrtodactylus* Gray, 1827 (Squamata: Gekkonidae) in Kien Glang Biosphere Reserve, Southwestern Vietnam. *Zootaxa*, 1967, 53–62.

Ngo, V. T., Grismer, L. L. & Grismer, J. L. (2010). A new species of *Cyrtodactylus* Gray, 1827 (Squamata: Gekkonidae) in Phu Quoc National Park, Kien Glang Biosphere Reserve, southwestern Vietnam. *Zootaxa*, 2604, 37–51.

Ngo, V. T. & Pauwels, O. S. G. (2010). A new cave-dwelling species of *Cyrtodactylus* Gray, 1827 (Squamata: Gekkonidae) from Khanmouane province, southern Laos. *Zootaxa*, 2730, 44–56.

Nguyen, T. Q., Kingsada, P., Rösler, H., Auer, M., & Ziegler, T. (2010). A new species of Cyrtodactylus (Squamata: Gekkonidae) from northern Laos. *Zootaxa* 2652, 1–16.

Nguyen, S. N., Le, T.-N. T., Tran, T. A D., Orlov, N. L., Lathrop, A. M. Y., Macculloch, R. D., Le, T.-D. T., Jin, J.-Q., Nguyen, L. T., Nguyen, T. T., Hoang, D. D., Che, J., Murphy, R. W. & Zhang, Y.-P. (2013). Phylogeny of the *Cyrtodactylus irregularis* species complex (Squamata: Gekkonidae) from Vietnam with the description of two new species. *Zootaxa*, 3737, 399–414.

Nguyen, T.Q., Le, M.D., Pham, A.V., Ngo, H.N., Hoang, C.V., Pham, C.T., & Ziegler, T. (2015). Two new species of *Cyrtodactylus* (Squamata: Gekkonidae) from the karst forest of Hoa Binh Province, Vietnam. Zootaxa, 3985, 375–390.

Nguyen, S. N., Orlov, N. L., & Darevsky, I. S. (2006). Descriptions of two new species of the genus *Cyrtodactylus* Gray, 1827 (Squamata: Sauria: Gekkonidae) from southern Vietnam. *Journal of Herpetology*, 13, 215–226.

Nguyen, T. Q., Pham, A. V., Ziegler, T., Ngo, T. H., & Le, M. D. (2017). A new species of *Cyrtodactylus* (Squamata: Gekkonidae) and the first record of *C. otai* from Son La Province, Vietnam. *Zootaxa,* 4341, 25–40.

Nguyen, S. N., Yang, J. X., Le, T. N., Nguyen, L. T., Orlov, N. L., Hoang, C. V., Nguyen, T. Q., Jin, J. Q., Rao, D. Q., Hoang, T. N., Che, J., Murphy, R. W. & Zhang, Y. P. (2014). DNA barcoding of Vietnamese bent-toed geckos (Squamata: Gekkonidae: *Cyrtodactylus*) and the description of a new species. *Zootaxa*, 3784, 48–66.

Nielsen, S. V., & Oliver, P. M. (2017). Morphological and genetic evidence for a new karst specialist lizard from New Guinea (*Cyrtodactylus*: Gekkonidae) *Royal Society Open Science,* 2017 4 170781; DOI: 10.1098/rsos.170781

O’Connell, K. A., Smart, U., Sidik, I., Riyanto, A., Kurniawn, N., & Smith, E. (2019). Diversification of bent-toed geckos (*Cyrtodactylus*) on Sumatra and west Java. *Molecular Phylogenetics and Evolution,* 134, 1–11.

Oliver, P., Krey, K., Mumpuni, & Richards, S. (2011). A new species of bent-toed gecko (Cyrtodactylus, Gekkonidae) from the North Papuan Mountains. *Zootaxa*, 2930, 22–32.

Oliver, M. P., Richards, S. J., Mumpuni, Rösler, H. (2016). The knight and the king: two new species of giant bent-toed gecko (*Cyrtodactylus,* Gekkonidae, Squamata) from northern New Guinea, with comments on endemism in North Papuan Mountains. *Zookeys,* 562, 105–130.

Quang, L. V., Calame, T., Bonkowski, M., Nguyen, T. Q., & Ziegler, T. (2014). A new species of *Cyrtodactylus* (Squamata: Gekkonidae) from Khammouane Province, Laos. *Zootaxa*, 3760, 54–66.

Quang, L. V., Calame, T., Bonkowski, M., Nguyen, T. Q., & Ziegler, T. (2015). A new species of *Cyrtodactylus* (Squamata: Gekkonidae) from the limestone forest of Khammouane Province, central Laos. *Zootaxa*, 4058, 388–402.

Quang, L. V., Nguyen, T. Q., Do, H. Q., & Ziegler, T. (2011). A new *Cyrtodactylus* (Squamata: Gekkonidae) from Huong Son limestone forest, Hanoi, northern Vietnam. *Zootaxa*, 3129, 39–50.

Quang, L. V., Nguyen, T. Q., Le, M. D., Bonkowski, M., Ziegler, T. (2016). A new species of karst-dwelling bent-toed gecko (Squamata: Gekkonidae) from Khammouane Province, central Laos. *Zootaxa* 4079, 87–102.

Luu, V. Q., Dung, V. T., Nguyen, T. Q., Le, M. D., & Ziegler, T. (2017). A new species of the *Cyrtodactylus irregularis* complex (Squamata: Gekkonidae) from Gia Lai Province, Central Highlands of Vietnam. *Zootaxa* 4362, 385–404.

Mahony, S., Hossain, Ahmed M., Hossain, M. K., Kabir, M. M., & Hasan, M. K. (2009). *Cyrtodactylus ayeyarwadyensis* Bauer, 2003 (Squmata: Gekkonidae) in Bangladesh with habitat details of new collection localities and a discussion of morphological variation. *Salamandra,* 45, 245–250.

Mirza, Z. A., Pal, S., & Sanap, R. V. (2010). Notes on a ground gecko *Geckoella* cf. *collegalensis* Beddome, 1870 (Squamata, Sauria, Gekkonidae) from India. *Russian Journal of Herpetology,* 17, 8–14.

Murdoch, M. L., Grismer, L. L., Wood, Jr., P. L., Thy, N., Poyarkov, N. A., Ngo, V. T., Nazarov, R. A., Aowphol, A., Pauwels, O. S. G., Nguyen, H. C., & Grismer, J. L. (2019). Six new species of the *Cyrtodactylus* intermedius complex (Squamata: Gekkonidae) from the Cardamom Mountains and associated highlands of Southeast Asia. *Zootaxa* 4554, 001–062.

Shea, Gl., Couper, P., Wilmer, J. W., & Amey, A. (2011). Revision of the genus *Cyrtodactylus* Gray, 1827 (Squamata: Gekkonidae) in Australia. *Zootaxa* 3146, 1–63.

Oliver, P. M, Richards, S. J., Mumpuni, & Rösler, H. (2016). The Knight and the King: two new species of giant bent- toed gecko (*Cyrtodactylus*, Gekkonidae, Squamata) from northern New Guinea, with comments. *ZooKeys*, 562, 105–130.

Oliver, P. M., Krey, K., Mumpuni, & Richards, S. J. (2011). A new species of bent-toed gecko (*Cyrtodactylus*: Gekkonidae) from the North Papuan Mountains. *Zootaxa*, 2930, 22–32.

Oliver, P. M., Tjaturadi, B., Mumpuni, Krey, K. & Richards, S. J. (2008). A new species of large *Cyrtodactylus* (Squamata: Gekkonidae) from Melanesia. *Zootaxa*, 1894, 59–68.

Oliver, P. M., Richards, S. J. & Sistrom, M. (2012). Phylogeny and systematics of Melanesia’s most diverse gecko lineage (*Cyrtodactylus*, Gekkonidae, Squamata). *Zoologica Scripta*, 41, 437-454.

Orlov, N. L., Nguyen, Q. T., Nazarov, R., Ananjeva, N. B., & Nguyen, N. S. (2007). A new speceis of the genus *Cyrtodactylus* Gray, 1827 and redecription of *Cyrtodactylus paradoxus* (Darevsky et Szczerbak, 1997) [Squamata: Sauria: Gekkonidae] from South Vietnam. *Russian Journal of Herpetology*, 14, 145–152.

Panitvong, N., Sumontha, M., Tunprasert, J. & Pauwel, O. S. G. (2014). *Cyrtodactylus saiyok* sp. nov., a new dry evergreen forest-dwelling Bent-toed Gecko (Squamata: Gekkonidae) from Kanchanaburi Province, western Thailand. *Zootaxa*, 3869, 64–74.

Pauwels, O. S. G., Bauer, A. M., Sumontha, M., & Chanhome, L. (2004). *Cyrtodactylus thirakhupti* (Squamata: Gekkonidae), a new cave-dwelling gecko from southern Thailand. *Zootaxa*, 772, 1–11.

Pham, A. V., Le, M. D., Ziegler, T., Nguyen, T. Q. (2019). A new species of *Cyrtodactylus* (Squamata: Gekkonidae) from northwestern Vietnam. *Zootaxa*, 4544, 360–380.

Purkaystha, J. Das, M. Bohra, S.C., Bauer, A.M., & Agarwal, I. (2020). Another new *Cyrtodactylus* (Squamata: Gekkonidae) from Guwahati, Assam, India. *Zootaxa,* 375–392.

Quah, E. S. H., Grismer, L. L., Wood, Jr., Sah, S. A. M. (2019). The discovery and description of a new species of Bent-toed Gecko of the *Cyrtodactylus pulchellus* complex (Squamata: Gekkonidae) from the Langkawi Archipelago, Kedah, Peninsular Malaysia. *Zootaxa,* 4668, 51–75.

Quang, H. X., Orlov, N. L., Ananjeva, N. B., Johns, A. G., Thao, H. N., & Vinh, D. Q. (2007). Description of a new species of the genus *Cyrtodactylus* Gray, 1827 (Squamata: Sauria: Gekkonidae) from the Karst of north central Vietnam. *Russian Journal of Herpetology,* 14, 98–106.

Riedel, J., Nordberg, E., & Schwarzkopf, L. (2020). Ecological niche and habitat use of an Australian gecko assemblage. *Israel Journal of Ecology & Evolution,* DOI:

<https://doi.org/10.1163/22244662-bja10002>

Riyanto, A., Grismer, L. L., & Wood, Jr., P. L. (2015b). *Cyrtodactylus rosichonariefi* sp. nov. (Squamata: Gekkonidae), a new swamp-dwelling bent-toed gecko from Bunguran Island (Great Natuna), Indonesia. *Zootaxa,* 3964, 114–124.

Riyanto, A., Grismer, L. L., & Wood, Jr., P. L. (2015a). The fourth Bent-toed Gecko of the genus *Cyrtodactylus* (Squamata: Gekkonidae) from Java, Indonesia *Zootaxa,* 4059, 351–363.

Rösler, H., Richards, S. J., & Günther, R. (2007). Remarks on morphology and taxonomy of geckos of the genus *Cyrtodactylus* Gray, 1827, occurring east of Wallacea, with descriptions of two new species (Reptilia: Sauria: Gekkonidae). *Salamandra*, 43, 193–230.

Scheider, N., Nguyen, T.Q., Schmitz, A., Kingsada, P., Auer, M. & Ziegler, T. (2011). A new species of karst dwelling *Cyrtodactylus* (Squamata: Gekkonidae) from northwestern Laos. *Zootaxa*, 2930, 1–21.

Schneider, N., Nguyen, T. Q., Le, M. D., Nophaseud, L., Bonkowski, M. & Ziegler, T. (2014). A new species of *Cyrtodactylus* (Squamata: Gekkonidae) from the karst forest of northern Laos. *Zootaxa*, 3835, 80­–96.

Shea, G., Couper, P., Wilmer, J. W., & Andrew, A. (2011). Revision of the genus *Cyrtodactylus* Gray, 1827 (Squamata: Gekkonidae) in Australia. *Zootaxa*, 3146, 1–63.

Shi, L. & Zhao, H. (2010). A new species of *Cyrtodactylus* (Reptilia: Squamata: Geckkonidae) from Xizang Autonomous Region, China. *Zootaxa*, 2336, 51–60.

Smith, M. J., Cogger, H., Tiernan, B., Maple, D., Boland, C., Napier, F., Detto, T. & Smith, P. (2012). An oceanic island reptile community under threat: the decline of reptiles on Christmas Island, Indian Ocean. *Herpetological Conservation and Biology*, 7, 206–218.

Sumontha, M, Kunya, K and Pauwels, O. S. G. (2008). Jarujin’s bent toed gecko

(*Cyrtodactylus jarujini*) in nature. *Ecological Notes. The Record of Nature*, 2, 22–23.

Taylor, E. H. (1963). The lizards of Thailand. *The University of Kansas Science Bulletin.*, 44, 687–1077.

Trung, L. D., Nguyen, T. Q., Le, M. D., Ziegler, T. (2016). A new species of *Cyrtodactylus* (Squamata: Gekkonidae) from Ninh Binh Province, Vietnam. *Zootaxa* 4162, 268–282.

Welton, L. J., Siler, C. D., Diesmos, A., & Brown, R. M. (2009). A new bent-toed gecko (Genus *Cyrtodactylus*) from Southern Palawan Island, Philippines and clarification of the taxonomic status of *C. annulatus*. *Herpetologica*, 65, 328–343.

Welton, L. J., Siler, C. D., Diesmos, A. & Brown, R. M. (2010a) Phylogeny-based species delimitation of southern Philippines bent-toed geckos and a new species of *Cyrtodactylus* (Squamata: Gekkonidae) from western Mindanao and the Sulu Archipelago. *Zootaxa*, 2390, 49–68.

Welton, L. J., Siler, C. D., Linkem, C. W., Diesmos, A. C. & Brown, R. M. (2010b) Philippine bent-toed geckos of the *Cyrtodactylus agusanensis* complex: multilocus phylogeny, morphological diversity, and descriptions of three new species. *Herpetological Monographs*, 24, 55–85.

Wood, Jr., P. L., Grismer, L. L., Muin, M. A., Anuar, S. Oaks, J. R., & Sites, Jr., J. W. (2020). A new potentially endangered limestone-associated Bent-toed Gecko of the *Cyrtodactylus pulchellus* (Squamata: Gekkonidae) complex from northern Peninsular Malaysia. *Zootaxa,* 4751, 437–460.

Worthington, J. & Wilmer, A. B., & Couper, P. (2015). Phylogeography of north-eastern Australia’s *Cyrtodactylus* radiation: a habitat switch highlights adaptive capacity at a generic level. *Australian Journal of Zoology, 63,* 398–410.

Youmans, T. M. & Grismer, L. L. (2006). A new species of *Cyrtodactylus* (Reptilia: Squamata: Gekkonidae) from the Seribaut Archipelago, West Malaysia *Herpetological Natural History*, 10, 61–70.

Yuan, S. Q. & Rao, D. Q. (2011). A new record of a gekkonid (*Cyrtodactylus wayakonei*) from Yunnan, China. *Zoological Research*, 32, 684–688.

Ziegler, T., Phung, T. M., Le, M. D. & Nguyen, T.Q. (2013). A new *Cyrtodactylus* (Squamata: Gekkonidae) from Phu Yen Province, southern Vietnam. *Zootaxa*, 3686, 432–446.
